# Immune response of BV-2 microglial cells is impacted by peroxisomal beta-oxidation

**DOI:** 10.3389/fnmol.2023.1299314

**Published:** 2023-12-18

**Authors:** Ali Tawbeh, Quentin Raas, Mounia Tahri-Joutey, Céline Keime, Romain Kaiser, Doriane Trompier, Boubker Nasser, Emma Bellanger, Marie Dessard, Yannick Hamon, Alexandre Benani, Francesca Di Cara, Tânia Cunha Alves, Johannes Berger, Isabelle Weinhofer, Stéphane Mandard, Mustapha Cherkaoui-Malki, Pierre Andreoletti, Catherine Gondcaille, Stéphane Savary

**Affiliations:** ^1^Laboratoire Bio-PeroxIL EA7270, University of Bourgogne, Dijon, France; ^2^Laboratory of Biochemistry, Neurosciences, Natural Resources and Environment, Faculty of Sciences and Techniques, University Hassan I, Settat, Morocco; ^3^Plateforme GenomEast, IGBMC, CNRS UMR 7104, Inserm U1258, University of Strasbourg, Illkirch, France; ^4^Aix Marseille Univ, CNRS, INSERM, CIML, Marseille, France; ^5^Centre des Sciences du Goût et de l'Alimentation, CNRS, INRAE, Institut Agro, University of Bourgogne, Dijon, France; ^6^Department of Microbiology and Immunology, Dalhousie University, IWK Health Centre, Halifax, NS, Canada; ^7^Department of Pathobiology of the Nervous System, Center for Brain Research, Medical University of Vienna, Vienna, Austria; ^8^LipSTIC LabEx, University of Bourgogne, INSERM LNC UMR1231, Dijon, France

**Keywords:** adrenoleukodystrophy, antigen presentation, immune response, inflammation, microglia, peroxisome, phagocytosis

## Abstract

Microglia are crucial for brain homeostasis, and dysfunction of these cells is a key driver in most neurodegenerative diseases, including peroxisomal leukodystrophies. In X-linked adrenoleukodystrophy (X-ALD), a neuroinflammatory disorder, very long-chain fatty acid (VLCFA) accumulation due to impaired degradation within peroxisomes results in microglial defects, but the underlying mechanisms remain unclear. Using CRISPR/Cas9 gene editing of key genes in peroxisomal VLCFA breakdown (*Abcd1, Abcd2*, and *Acox1*), we recently established easily accessible microglial BV-2 cell models to study the impact of dysfunctional peroxisomal β-oxidation and revealed a disease-associated microglial-like signature in these cell lines. Transcriptomic analysis suggested consequences on the immune response. To clarify how impaired lipid degradation impacts the immune function of microglia, we here used RNA-sequencing and functional assays related to the immune response to compare wild-type and mutant BV-2 cell lines under basal conditions and upon pro-inflammatory lipopolysaccharide (LPS) activation. A majority of genes encoding proinflammatory cytokines, as well as genes involved in phagocytosis, antigen presentation, and co-stimulation of T lymphocytes, were found differentially overexpressed. The transcriptomic alterations were reflected by altered phagocytic capacity, inflammasome activation, increased release of inflammatory cytokines, including TNF, and upregulated response of T lymphocytes primed by mutant BV-2 cells presenting peptides. Together, the present study shows that peroxisomal β-oxidation defects resulting in lipid alterations, including VLCFA accumulation, directly reprogram the main cellular functions of microglia. The elucidation of this link between lipid metabolism and the immune response of microglia will help to better understand the pathogenesis of peroxisomal leukodystrophies.

## Introduction

Microglia, the resident immune cells of the central nervous system, are crucial in regulating neural activity, synaptic plasticity, and neuroinflammation (Wright-Jin and Gutmann, 2019). Microglia play important roles in brain development and function, and alteration of their homeostatic state has been linked to various neurological disorders (Sirkis et al., 2021; Paolicelli et al., 2022). Microglia respond to injury and infection by releasing inflammatory mediators phagocytosing myelin debris and dying cells while promoting myelin repair and regeneration. Despite extensive research into their immune functions and signaling pathways, little attention has been paid to how organelles control the functions of microglia.

Using CRISPR/Cas9 genome editing, we recently engineered novel, easily accessible murine BV-2 models carrying mutations in peroxisomal genes (*Abcd1, Abcd2*, and *Acox1*) to study the impact of peroxisomal defects in microglial cells (Raas et al., 2019a,b). The final aim was to gain insight into the pathogenesis of peroxisomal leukodystrophies such as X-linked adrenoleukodystrophy (X-ALD, MIM 300100) and acyl-CoA oxidase 1 (ACOX1) deficiency (MIM 264470). X-ALD, the most frequent peroxisomal disease, is a complex neurodegenerative disorder with two major clinical forms: the childhood cerebral form (cALD), showing rapid progressive inflammatory cerebral demyelination, and the adult form called adrenomyeloneuropathy (AMN), showing a slowly progressive and non-inflammatory spinal cord disease. Independent of the variability of clinical symptoms, X-ALD is associated with mutations in the *ABCD1* gene, which encodes an ATP-binding cassette transporter of very long-chain fatty acids (VLCFAs) localized at the peroxisomal membrane (Mosser et al., 1993). The disease is characterized by impaired peroxisomal β-oxidation and VLCFA accumulation (Trompier and Savary, 2013; Kemp et al., 2016). In agreement with the functional redundancy between *Abcd1* and *Abcd*2, its closest homolog (Mosser et al., 1993; Lombard-Platet et al., 1996; Genin et al., 2011; Tawbeh et al., 2021), VLCFA accumulation was absent in the single knockouts of either *Abcd1* or *Abcd2* but was present in the *Abcd*1^−/−^*Abcd*2^−/−^ double knockout mutant BV-2 cells. The ACOX1 enzyme controls the first step of peroxisomal β-oxidation and its defect is associated with a very rare and severe leukodystrophy, resulting in the accumulation of VLCFA in plasma and tissues (Fournier et al., 1994; Nohammer et al., 2000; Ferdinandusse et al., 2007; Vamecq et al., 2018). Accordingly, *Acox*1^−/−^ BV-2 cells also demonstrate VLCFA accumulation, although less marked than in *Abcd*1^−/−^*Abcd*2^−/−^ cells. Furthermore, apart from the noted VLCFA-related findings and the crosstalks existing between peroxisomal ABC transporters and ACOX1, we also observed the accumulation of neutral lipids, alterations in membrane structure, the presence of lipid inclusions, and the occurrence of lipid droplets in the mutant cell lines. This was accompanied by a significant reprogramming of gene expression patterns associated with lipid metabolism that could be attributed in part to PPARβ activation, which is highly expressed in BV-2 microglial cells (Raas et al., 2023).

Despite some limitations, including a lower reactivity than primary cells upon LPS treatment (Das et al., 2016), the BV-2 cell line displays many microglial features, such as phagocytosis and the ability to respond to inflammatory stimulation through the secretion of nitric oxide (NO) and pro-inflammatory cytokines (Henn et al., 2009; Das et al., 2015). Thus, these cells serve as a useful tool and continue to be the most used cell line for studying microglial behavior and inflammatory response *in vitro*. Using the BV-2 cell models, we could demonstrate that a defect in peroxisomal fatty acid β-oxidation results in a reprograming of gene expression linked to lipid metabolism, lysosome, autophagy, and in the emergence of a disease-associated microglial-like (DAM-like) signature (Raas et al., 2023). Interestingly, some of the main hub genes of microglia (*Apoe, Tyrobp*, and *Trem2*), which play pivotal roles in modulating microglial functions, were found to be significantly upregulated in the knockout genotypes. TREM2, with its downstream adaptor TYROBP/DAP12, constitutes a receptor for diverse ligands, including LPS, that plays a key role in microglial differentiation, phagocytosis, and inflammatory responses (Jay et al., 2017; Krasemann et al., 2017; Gratuze et al., 2018; Ulland and Colonna, 2018; Haure-Mirande et al., 2022). Moreover, the gene ontology term “Innate immune response” was the most enriched term among the differentially expressed genes (DEGs) found in our mutant BV-2 cells.

Recent research has highlighted the physiological role of peroxisomes and their importance in the regulation of immune and inflammatory responses (Di Cara et al., 2019, 2023; Di Cara, 2020; Wanders et al., 2023). However, there is currently a lack of studies investigating the relationship between peroxisomes and microglia. Peroxisome proliferator-activated receptor delta (PPARδ)-deficient microglia were demonstrated to overexpress genes associated with phagocytosis and inflammation (Doroshenko et al., 2021). MFP2 deficiency, which also leads to a peroxisomal β-oxidation defect, was shown to provoke a microgliosis and a chronic inflammatory profile (Beckers et al., 2018, 2019). In X-ALD, defective microglia have been supposed to prime and amplify the neurodegenerative process (Gong et al., 2017; Bergner et al., 2019). Blood–brain barrier rupture, allowing the infiltration, recruitment, and activation of peripheral immune cells such as macrophages and T lymphocytes, would contribute to neuroinflammation and demyelination (Griffin et al., 1985; Ito et al., 2001; Zierfuss et al., 2022). Allogeneic hematopoietic stem cell transplantation and, more recently, cell-based gene therapy have been shown to halt disease progression (Cartier et al., 2009; Eichler et al., 2017). The exact mechanism by which this therapeutic strategy proves to be efficient in the brain is unclear, but the functional replacement of microglia by monocyte/macrophage cells derived from hematopoietic stem cells is suspected (Weinhofer et al., 2018). X-ALD macrophages were described as pro-inflammatory skewed and unable to complete anti-inflammatory response, suggesting that peroxisomal β-oxidation of VLCFAs is central in the resolution of inflammation (Weinhofer et al., 2018; Zierfuss et al., 2022). Immune system alterations, including macrophage and T cell activation, as well as cytokine production, may explain some of the variability observed in the clinical picture. In addition to gene mutations, environmental factors, including infections and other inflammatory stimuli, may also influence disease manifestation (Wiesinger et al., 2015). The clinical variability observed in X-ALD, even in identical twins (Korenke et al., 1996), suggests that other factors beyond genetic mutations contribute to the pathogenesis.

To investigate in more detail the functional consequences of the peroxisomal defects in BV-2 microglial cells related to immune response, we performed RNA-sequencing in WT and mutant cell lines and compared the response to a 24-h treatment with LPS. We investigated the inflammatory response of the *Abcd*1^−/−^*Abcd*2^−/−^ and *Acox*1^−/−^ BV-2 microglial cells by studying the expression and secretion of key inflammatory cytokines and chemokines. We explored the consequences of the peroxisomal β-oxidation defects on the phagocytic capacity of microglial cells. Finally, using calcium imaging, we studied the effect of peroxisomal dysfunction on microglial antigen presentation to CD8+ T cells, a crucial role in priming T lymphocyte response.

## Materials and methods

### Resource availability

The data discussed in this publication have been deposited in NCBI's Gene Expression Omnibus (Edgar et al., 2002) and are accessible through GEO Series accession number GSE200022 (https://www.ncbi.nlm.nih.gov/geo/query/acc.cgi?acc=GSE200022) for the genome comparison and GSE237635 for the LPS effect. The data supporting the findings and the excel files containing the gene lists used to create the figures are available from the corresponding author (SS) upon request.

### Cell culture

The mouse microglial BV-2 cell line was purchased from Banca-Biologica e Cell Factory (catalog no. ATL03001). Single or double mutant BV-2 cells, deficient for the peroxisomal proteins ACOX1 (*Acox1*^−/−^) or ABCD1 and ABCD2 (*Abcd1*^−/−^*Abcd2*^−/−^) were obtained by CRISPR/Cas9 editing (Raas et al., 2019a,b). Sanger sequencing confirmed the absence of CRISPR/Cas9-induced mutations in the two genomic sites with the highest predicted likelihood of off-target binding strongly, suggesting the absence of off-target events. WT and mutant BV-2 cells were grown in DMEM supplemented with 10% heat-inactivated FBS (Corning), 100 U/mL penicillin, and 100 μg/mL streptomycin (Gibco). Cultures were maintained at 37°C in a humidified atmosphere containing 5% CO_2_. WT and mutant BV-2 cells were seeded in 6-well plates at a density of 2 × 10^5^ cells/well. The day after, cells were treated for 24 h with 1 μg/mL LPS from *Escherichia coli* O55:B5 (Sigma-Aldrich). A vehicle containing 0.01% ethanol and 0.5 mM α-cyclodextrin was applied in each condition.

T lymphocytes were obtained from transgenic mice and maintained in DMEM F12 medium (Lonza). Animal experiments respected French and European directives. Animals were housed in cages with water and food *ad libitum* in the CIML animal house facility in Marseille. OT-1 mice specific for H-2Kb/ovalbumin (SIINFEKL) were kept on a *Rag*−1^−/−^ C57BL/6 background. Spleens and lymph nodes were recovered from heterozygous *Rag*1^+/−^ OT-1^+/−^ transgenic mice originating from the crossing of OT-1 mice with C57/Bl6 mice (Charles River). Organ dilacerations were done onto a nylon membrane in DMEM F12 medium (Lonza), and splenic erythrocytes were removed in Gey's lysis solution. CD8^+^ T cells were isolated by depleting the CD8 negative cells according to manufacturer instructions (Dynal Mouse CD8 Negative Isolation Kit, Invitrogen).

### RNA-sequencing, differential gene expression analysis, and bioinformatics

Total RNA was extracted from three independent batches of BV-2 cells for each genotype (WT, *Abcd1*^−/−^*Abcd2*^−/−^, *and Acox1*^−/−^) using an RNeasy kit (Qiagen). RNA-seq library preparation, sequencing, and analysis were performed as previously described (Raas et al., 2023). Differential gene expression analyses were performed using R 3.3.2 and DESeq2 version 1.16.1 Bioconductor library (Love et al., 2014). To analyze the effect of the treatment, we define a model with one factor (the corresponding condition). To test if the treatment effect differs between two genotypes, we define a model with two factors (genotype and treatment) and their interaction. Wald test *p*-values were adjusted for multiple testing using the Benjamini and Hochberg (1995) method. DEGs were selected using the following thresholds: adjusted *p*-value lower than 0.05 and absolute log2 Fold Change (FC) higher than 1. Venn diagrams were obtained using Venny 2.1 (https://bioinfogp.cnb.csic.es/tools/venny/index.html). Gene ontology analysis and gene set enrichment analysis were performed using ShinyGO online tools (http://bioinformatics.sdstate.edu/go/) (Ge et al., 2020). Expression-based heatmaps and hierarchical clustering were performed with Heatmapper (http://www.heatmapper.ca/) (Babicki et al., 2016), using average linking and Pearson distances.

### Analysis of protein expression by Western blotting

Cell lysate proteins (30 μg) were separated by SDS-PAGE and transferred to PVDF membranes. Membranes were first blocked in 5% fat-free milk in PBST (Phosphate buffer saline, 0.1% Tween 20) and then probed with primary antibodies in 1% fat-free milk in PBST. The following antibodies were used with the indicated dilution: anti-CASP1 (1:1,000, Adipogen AG-20B-0042), anti-CD36 (1:1,000, R&D Systems AF2519), anti-FCGR2B (1:1,000, Cell Signaling 96397), anti-IL1B (1:1,000, GeneTex GTX 74034), anti-MRC1 (1:1,000, Abcam ab64693), anti-NLRP3 (1:3,000, Adipogene AG-20B-0014), anti-TLR2 (1:500, R&D Systems AF1530), and anti-TLR4 (1:1,000, Santa Cruz sc-293072). Membranes were washed in PBST and incubated with the appropriate HRP-conjugated secondary antibodies (1:5,000) in 1% fat-free milk in PBST. After five washes, immunoreactivity was revealed by incubating membranes with the HRP SuperSignal West Femto Maximum Sensitivity Substrate (ThermoFisher Scientific), and the signal was detected by the Chemidoc XRS system (Bio-Rad). Protein amount loading control and normalization were achieved by probing the membranes with α-tubulin (TUBA1A) (1:4,000, Sigma-Merck T5168) or β-actin (ACTB) (1:10,000, Sigma-Merck A5441) antibodies.

### Analysis of protein expression by cytometry

BV-2 cell staining was performed in 1X PBS with 1% BSA after blocking the Fc receptors with 2.4G2 (BD Bioscience). Expression of H2-K1 (H2Kb, MHC I), CD80, CD86, and CD54 (ICAM) was assessed using the following antibodies: anti-H2-Kb AF647, clone AF6-88.5 (Biolegend), anti-CD80-PE, clone 16-10A1 (eBioscience), anti-CD86-PE, clone gl1 (eBioscience), anti-CD54-APC, and clone YN1/1.7.4 (Biolegend). Control experiments of autofluorescence with unlabeled cells and the use of an irrelevant antibody (isotype control) to measure background fluorescence were used in each experiment. Surface marker analysis was performed on an LSR II flow cytometer (Becton Dickinson) using the FACSDiva software. Data analysis was performed with FlowJo (Tree star).

### Multiplex analysis of cytokines

The WT and mutant BV-2 wells were seeded in two 6-well plates in 3 mL DMEM supplemented with 10% FBS and 1% penicillin-streptomycin antibiotics. Twenty-four hours later, the cells of one plate were treated with 1 μg/mL LPS, whereas the other was kept at basal conditions. After 48 h in culture, the supernatants of the BV-2 cells were collected and centrifuged at 300 g for 7 min and then stored at −80°C until used. On the day of the experiment, the cell culture supernatants were subjected to multiplex analysis using Milliplex Map kit #MCYTOMAG-70K according to manufacturers' instructions. Samples were tested under two different conditions (undiluted or diluted 50-fold) to meet the concentration range of the standard curve for all the cytokines being tested. For each of the dilution conditions, three biological replicates for each sample were tested. The immunoassay was performed using Bio-Plex 200 (Bio-Rad), equipped with Bio-Plex Manager for acquisition and data analysis.

### Myelin isolation and labeling

Myelin was isolated from the brain tissue of 6–8-week-old male wild-type C57BL/J6 mice by ultracentrifugation according to the protocols of Norton and Poduslo (1973) and Larocca and Norton (2007). The isolated myelin was recovered in PBS and tested for putative LPS contamination using the ToxinSensor TM Chromogenic LAL Endotoxin Assay kit (GenScript). The isolated myelin was fluorescently labeled by incubating 1 mg myelin in PBS with 10 μL pHrodo^®^-Red succinimidyl ester in DMSO (1 mg/mL, Life Technologies) for 45 min at room temperature in the dark. Excess dye was removed by short centrifugation, and the pHrodo-labeled myelin was resuspended in PBS (pH 7.4) and stored in aliquots at −20°C.

### Phagocytosis analysis of pHrodo™ green-labeled myelin and *E. coli* bioparticles

The phagocytic ability of BV-2 WT and KO cells was assessed using pHrodo™ Green-labeled myelin and *E. coli* BioParticles™ Conjugate (Thermofisher, P35366). Cells were seeded in 96-well plates and allowed to settle before being treated with LPS 1 μg/mL for 24 h. Cells were then incubated with 10 μg/mL labeled myelin or 25 μg/mL with fluorescent bioparticles. Afterward, the plates were transferred to the IncuCyte^®^ SX5 live-cell analysis system (Sartorius), kept in cell culture conditions (37°C and 5% CO_2_), and the fluorescent activity was recorded every 30 min for 16 h. Five replicates were included for each condition and three repetitions for each experiment were carried out. Data were analyzed and extracted using the IncuCyte^®^ software with the florescence normalized over the number of cells through the following defined metric: green integrated intensity per image/phase area confluence. The fluorescence background was systematically subtracted.

### Phagocytosis analysis of fluoresbrite^®^ yellow green carboxylate beads

BV-2 cells (100,000 cells/well) were plated in 12-well plates overnight for 24 h. Fluoresbrite^®^ Yellow Green (YG) carboxylate microspheres (1 μm) (Polysciences) were washed and plated on BV-2 cells at a cell/beads ratio of 1:200 at 37°C for 1 h. Phagocytosis was stopped by washing the cells with ice-cold PBS. Phagocytosis was immediately quantified on an LSRII flow cytometer (BD Biosciences), with the addition of Trypan blue 0.04% in PBS 1X to quench extracellular fluorescence from attached but not internalized YG beads. For negative controls, equivalent cell samples were pre-treated with the phagocytosis inhibitor cytochalasin-D (37°C, 4 μM, Biotrend), then kept on ice and maintained in 4 μM cytochalasin-D during cell exposure to YG beads. Data are expressed as the percentage of YG beads-positive cells (i.e., cells that have ingested at least two beads) and as the phagocytic index (PI) calculated using the following formula: PI = (% YG beads-positive cells) × (Mean Fluorescence Intensity of YG beads-positive cells). Data were analyzed using the FlowJo X 10.0.7 software.

### Barcoding T cell calcium response

#### Loading protocol

10^6^ primary OT1 CD8+ T cells per well were plated in 96 plates in 100 μL of DMEM F12 (Gibco) and Nutridoma-SP 1% (Roche diagnostic). Cells were loaded with BD PBX diluted in 1X dye loading solution (according to manufacturer instructions) at 1/1,666 for primary T cells, i.e., ~0.6 μM. Then, 100 μL of this solution was dispatched to each well before incubation at 37°C for 30 min in the dark. Cells were washed twice in Hank's balanced salt solution (HBSS) Hepes 0.01M and resuspended in the same medium. Five wells were pooled (5.10^6^ cells) and seeded onto BV-2 cells overnight treated with LPS (LPS from *E.coli* 055: B5, Sigma, 1 μg/mL), then loaded for 1 h with various concentrations of the OVA SIINFELKL peptide (Eurogentec) and analyzed by live imaging microscopy.

#### Acquisition procedure

Movies were realized on a Zeiss LSM 780 confocal microscope. Pictures were taken with a C-Apochromat 40X/1.4 oil immersion objective, using an argon laser at 488 nm dichroic and a 505-nm long pass filter at 37°C using a hot plate. During acquisition, T cells were added to the medium containing BV-2 as antigen-presenting cells. Time-lapse movies are composed of 600 images taken every 6 s.

#### Analytical parameters

The cell tracking and the calcium signal analysis were achieved by the MAAACS software, as previously described (Salles et al., 2013). All parameters obtained in calcium experiment analysis (fluorescence amplitude and percentage of responding cells) were adapted for naïve CD8+ T cells by taking into consideration a 1.7 threshold level upon optimizing the product of their probability of detection times their probability of false alarm [PD × (1-PFA)] as mentioned by Salles et al. (2013). Analysis of calcium signals is represented in a dot plot only for cells that respond specifically (above threshold). Each point corresponds to the value of a parameter defined above.

#### Statistical analysis

All statistical analyses were performed using GraphPad Prism 9.4.

### Statistical analyses

The different statistical tests which were applied are indicated in the figure legends.

## Results

### Global analysis of the LPS response

**Figure 1:**
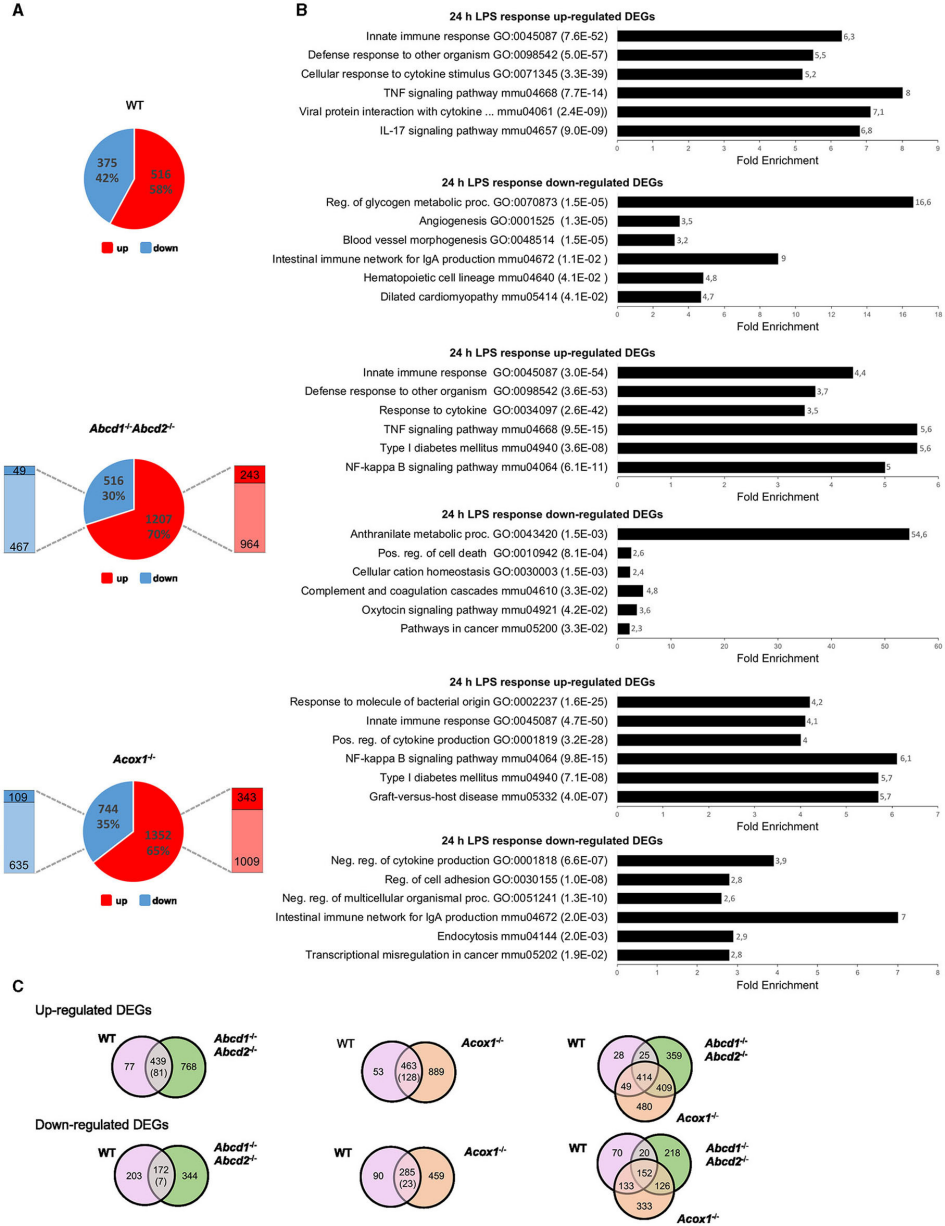
Transcriptomic analysis of the LPS response of WT and mutant (*Abcd*1^−/−^*Abcd*2^−/−^ and *Acox*1^−/−^) BV-2 cells (*n* = 3 for each genotype). **(A)** Pie chart displaying the number of up- or downregulated DEGs after a 24-h LPS treatment (DataSet GSE237635). The significant genes (number and percentage) were selected using a cutoff adjusted *p*-value (DESeq2 Wald test with Benjamini and Hochberg *p*-value adjustment) lower than 0.05 and an absolute log2 FC higher than 1. Additional comparison to test if the effect of the LPS treatment is different between the mutant and the WT genotypes, which permitted to highlight the proportion of significantly (AdjP Value < 0.05) more repressed (dark blue) (Log2FC < 1) or induced (dark red) (Log2FC > 1) DEGs, indicated by the left and right bars, respectively. **(B)** Gene set enrichment analysis in each genotype presenting a selection of the three main gene ontology biological process (up) and KEGG pathway (down) terms selected by FDR (in brackets) and sorted by Fold Enrichment (abscissa axis). **(C)** Comparative analysis of the sets of DEGs using the Venn diagram illustrating the larger increase of upregulated (up) and downregulated (down) DEGs in LPS-treated mutant cells as compared to WT. The number in brackets represents the number of DEGs belonging to the intersection significantly more deregulated (*p* < 0.05) in the mutant than in the WT genotype (Wald test).

One notable finding from the transcriptomic analysis of the *Abcd*1^−/−^*Abcd*2^−/−^ and *Acox*1^−/−^ mutant BV-2 cell lines was that the term “Innate immune response” consistently ranked as the top hit across all mutant genotypes (Raas et al., 2023). Genes encoding cytokines and chemokines, defense receptors such as Toll-like receptors (TLRs), proteins involved in immune signaling, response to viruses or bacteria, and inflammasome figured among the DEGs. *Ighm, C1rb, Nlrx1, Grap, Grap2, Il23, Cd14*, and *Nlrp3* were among the most downregulated genes, and *Rsad2, Clec4n, Nlrc3, Ifit3*, and *Tlr1* were among the most upregulated DEGs (Raas et al., 2023). These changes in gene expression may suggest that the ability of microglial cells to respond to immune stimuli has been altered. Genes encoding pro-inflammatory cytokines (*Il6* and *Tnf)* and the main actor of inflammasome (*Nlrp3*) were mostly repressed. Paradoxically, genes encoding pro-inflammatory chemokines (*Ccl3* and *Ccl4*) were induced in *Abcd*1^−/−^*Abcd*2^−/−^, and the gene encoding interleukin 1 beta (*Il1b*) was induced in *Acox*1^−/−^. When we compared the transcriptional response of WT and mutant BV-2 cells treated for 24 h with LPS (1 μg/mL), we found 891 genes significantly and differentially expressed in the WT cells, while 1,723 and 2,096 DEGs were revealed in the *Abcd*1^−/−^*Abcd*2^−/−^ and *Acox*1^−/−^, respectively, possibly indicating a stronger response to pro-inflammatory stimulation in peroxisomal mutants than in control cells (Figure 1A). Of the genes upregulated by LPS, 243 (20.1%) and 343 (25.4%) were significantly more upregulated in mutant genotypes than in WT. Similarly, 49 (9.5%) and 109 (14.7%) of repressed DEGs were significantly more downregulated in *Abcd*1^−/−^*Abcd*2^−/−^ and *Acox*1^−/−^ genotypes, respectively, when compared to WT (Figure 1A).

### LPS response and inflammatory cytokines

Enrichment analysis of the upregulated DEGs revealed quite similar results between genotypes for the first hits in link with immune response and pro-inflammatory response (innate immune response, response to cytokine, defense response to other organisms for GO terms; TNF signaling pathway, NF-κB signaling pathway, cytokine-cytokine receptor interaction, and NOD-like receptor signaling pathway for KEGG pathways). Of note, “NF-kappa B signaling” came out in a higher position in the KEGG enrichment for mutant genotypes, likely illustrating the amplificated response of mutant cell lines (Figure 1B). Among the downregulated DEGs, enrichment results were, however, divergent between genotypes. Venn diagrams illustrate a common response to LPS stimulation and that about one-third of the DEGs were exclusive to one single mutant genotype (Figure 1C). Statistical analysis of the LPS response showed that 18% (81/439) and 28% (128/463) of DEGs were significantly more upregulated in *Abcd*1^−/−^*Abcd*2^−/−^ and *Acox*1^−/−^ than in WT cells, respectively. In the downregulated genes, 4% (7/172) and 8% (23/285) of the downregulated DEGs were found significantly more repressed in *Abcd*1^−/−^*Abcd*2^−/−^ and *Acox*1^−/−^ genotypes, respectively. Altogether, these data indicate a stronger response both in the number of DEGs and in fold change in mutant cells impaired for peroxisomal β-oxidation.

As expected from previous studies in WT BV-2 cells (Lund et al., 2006; Henn et al., 2009; Das et al., 2015, 2016), LPS treatment resulted in the upregulation of pro-inflammatory genes encoding cytokines (*Tnf* , *Il1b, Il6, …*), chemokines (*Ccl3, Ccl4, Ccl5, Cxcl2, Cxcl*3,...), transcription factors (*Nfkb1* and *Nfkb2*), and various markers of microglial activation such as *Nos2* along with the downregulation of *Arg1*. All of these genes are figured in the intersection list of the three genotypes, illustrating similar LPS responses in regard to these genes between WT and mutant genotypes (Figure 1C).

**Figure 2:**
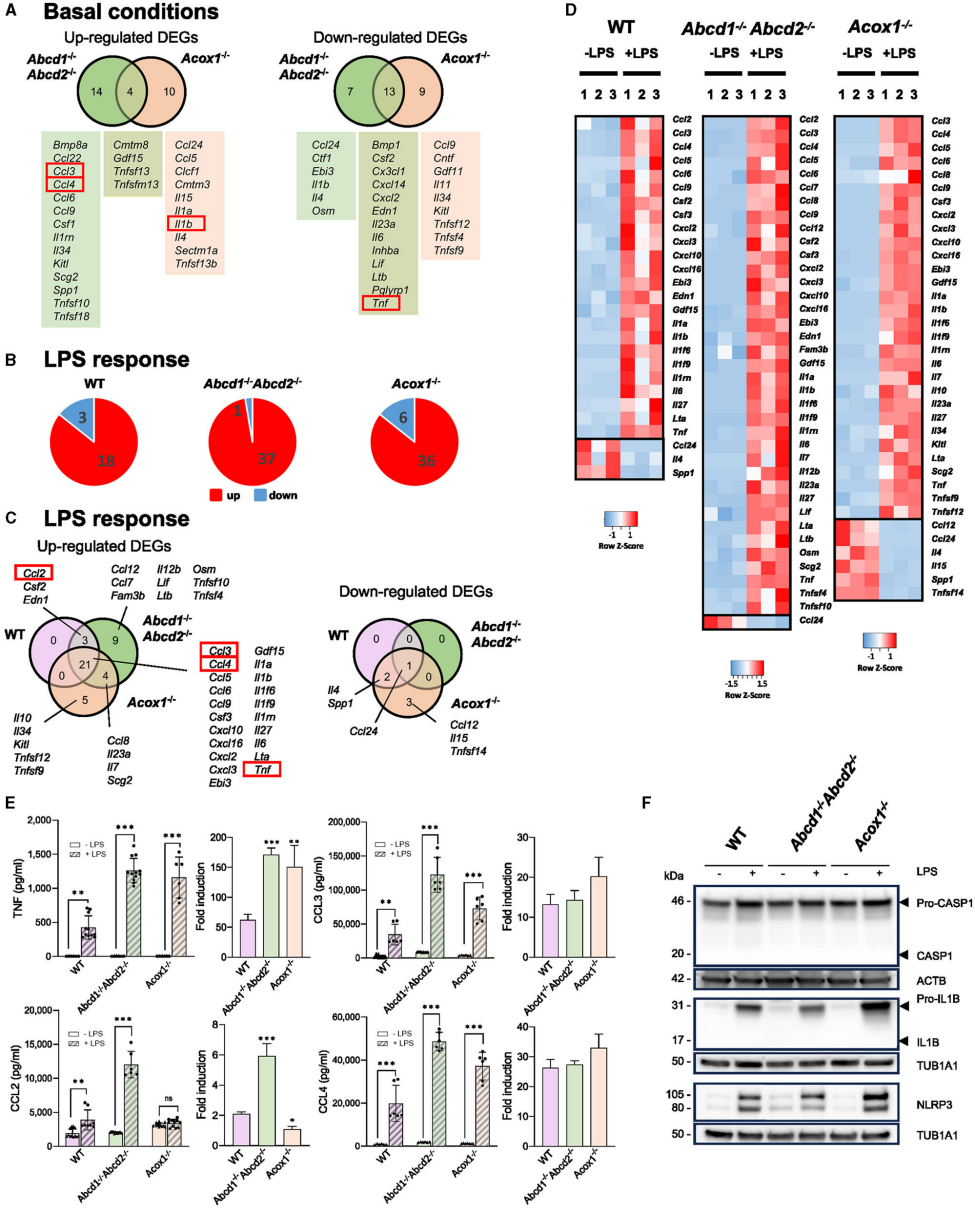
Impact of peroxisomal defect on the expression of cytokine and chemokine encoding genes in WT and mutant (*Abcd*1^−/−^*Abcd*2^−/−^ and *Acox*1^−/−^) BV-2 cells. **(A)** Venn diagrams illustrating the up- and downregulated cytokine and chemokine encoding genes DEGs (Log2FC > 1, AdjP Value < 0.05) *in Abcd*1^−/−^*Abcd*2^−/−^ and *Acox*1^−/−^ non-stimulated BV-2 cells (DataSet GSE200022). Genes whose expression level was measured at the protein level are boxed. **(B)** Pie chart displaying the number of these up- or downregulated DEGs after a 24-h LPS treatment (Log2FC > 1, AdjP Value < 0.05) in *WT, Abcd*1^−/−^*Abcd*2^−/−^, and *Acox*1^−/−^ BV-2 cells (DataSet GSE237635). **(C)** Venn diagrams representing the LPS response of these DEGs in each genotype. **(D)** Heat map representing RNA-Seq gene expression (with *Z*-scores) of these DEGs at 24 h after LPS stimulation in WT, *Abcd*1^−/−^*Abcd*2^−/−^, and *Acox*1^−/−^ BV-2 cells (three independent batches of BV-2 cells for each genotype). **(E)** Multiplex analysis of the concentration of secreted cytokines and chemokines (CCL2 (MCP-1), CCL3 (MIP-1α), CCL4 (MIP-1β), and TNF). Dot plots histograms present the single values and mean (±SD) of 6–12 measurements in the absence of LPS (empty bars) or after a 24-h treatment with LPS (hatched bars). Statistical differences relative to the untreated genotype are indicated (two-way ANOVA, *p < 0.01, **p < 0.01, ***p < 0.001). Relative fold induction is presented on the right histogram for each cytokine, and statistical differences relative to the fold induction observed in WT cells are indicated (one-way ANOVA, *p < 0.01, **p < 0.01, ***p < 0.001). **(F)** Representative image of Western blotting analysis (three independent experiments) of the expression levels of Caspase 1 (CASP1 and Pro-CASP1), Interleukin 1β (IL1B and Pro-IL1B), and NLRP3 in WT, *Abcd*1^−/−^*Abcd*2^−/−^, and *Acox*1^−/−^ BV-2 cells untreated (–) or treated with LPS for 24 h (+). Expected molecular weights are indicated. Source data are available online for this figure.

The secretion of cytokines and chemokines is considered one of the main immunomodulatory functions of microglial cells, directly affecting neurons. In basal conditions, we previously noticed that the *Abcd*1^−/−^*Abcd*2^−/−^ and *Acox*1^−/−^ mutants present a different expression pattern at the mRNA level for several cytokine-encoding genes (Raas et al., 2019b, 2023). Regarding the apparent amplified LPS response of mutant genotypes at the mRNA level, it was, therefore, important to pay attention to this group of genes. RNA-seq analysis confirmed that cytokine and chemokine encoding genes figure among the DEGs both in the absence and presence of LPS. In basal conditions, *Ccl3* and *Ccl4* genes were specifically upregulated in the *Abcd*1^−/−^*Abcd*2^−/−^ cells, and *Il1b* was found upregulated in the *Acox*1^−/−^ cells. *Tnf* and *Il6* genes were found downregulated in the *Abcd*1^−/−^*Abcd*2^−/−^ and *Acox*1^−/−^ cells (Figure 2A). The LPS treatment mainly resulted in the upregulation of pro-inflammatory genes, with mutant genotypes displaying a more important number of induced genes (Figure 2B). Venn diagram illustrating the LPS response shows that the largest group of upregulated DEGs is in the intersection of the three genotypes, which includes *Ccl3, Ccl4, Il1b, Il6*, and *Tnf* (Figures 2C, D). The *Ccl2* gene was upregulated in the WT and *Abcd*1^−/−^*Abcd*2^−/−^ genotypes only. The upregulation of these genes was found to be significantly increased in the mutants compared to the WT cells. Of note, both the inflammasome *Nlrp3* gene and the non-canonical inflammasome *Casp4* gene (Casson et al., 2015) were overexpressed upon LPS treatment, their induction being significantly higher in the mutants than in the WT BV-2 cells.

To confirm these results and check whether the secretion of these pro-inflammatory chemokines and cytokines is increased, we performed Bio-Plex analysis to measure the secretion of CCL2 (MCP-1), CCL3 (MIP-1α), CCL4 (MIP-1β), IL1B, and TNF. LPS treatment resulted in a significantly increased secretion of CCL3, CCL4, and TNF, regardless of the genotype; the CCL2 level was also significantly increased except in the *Acox*1^−/−^ cells (Figure 2E). The highest concentrations of the four analyzed cytokines were found in the supernatant of the *Abcd*1^−/−^*Abcd*2^−/−^ cells. However, WT and mutant BV-2 cells were unable to secrete IL1B, even upon LPS treatment. Interestingly, if we analyze the levels of the cytokines in a single genotype, the LPS-associated increase in TNF secretion was significantly higher in the mutant genotypes (Figure 2E). The LPS-associated increase in CCL2 secretion was also significantly higher in the *Abcd*1^−/−^*Abcd*2^−/−^ genotype (Figure 2E). These significant LPS-dependent over-secretion were not detected for CCL3 and CCL4 (Figure 2E).

As no IL1B secretion was detected, we wondered whether there was a maturation problem and also investigated the expression of the NLRP3/CASP1 inflammasome complex, which controls the release of IL1B and IL18 (Li and Jiang, 2023). Western blotting analysis confirmed the increase in pro-IL1B expression upon LPS treatment in each genotype but the absence of maturation of the protein (Figure 2F). The pro-IL1B signal was higher in the *Acox*1^−/−^ cell lines than in the control (Figure 2F). This maturation depends on the activation of caspase 1 (*Casp1*) by ASC (*Pycard*). Expression of pro-CASP1 was high and remained stable upon LPS treatment, but CASP1 activation was not detected (Figure 2F), which is likely explained by a very low level of *Pycard* expression in BV-2 cells, rendering impossible maturation and secretion of IL1B. However, we measured the expression of NLRP3 and confirmed its increased levels in each genotype upon LPS treatment, with higher signal intensity in the *Acox*1^−/−^ cells (Figure 2F). A densitometric analysis of the blots is presented in Supplementary Figure 1.

### Phagocytosis

**Figure 3:**
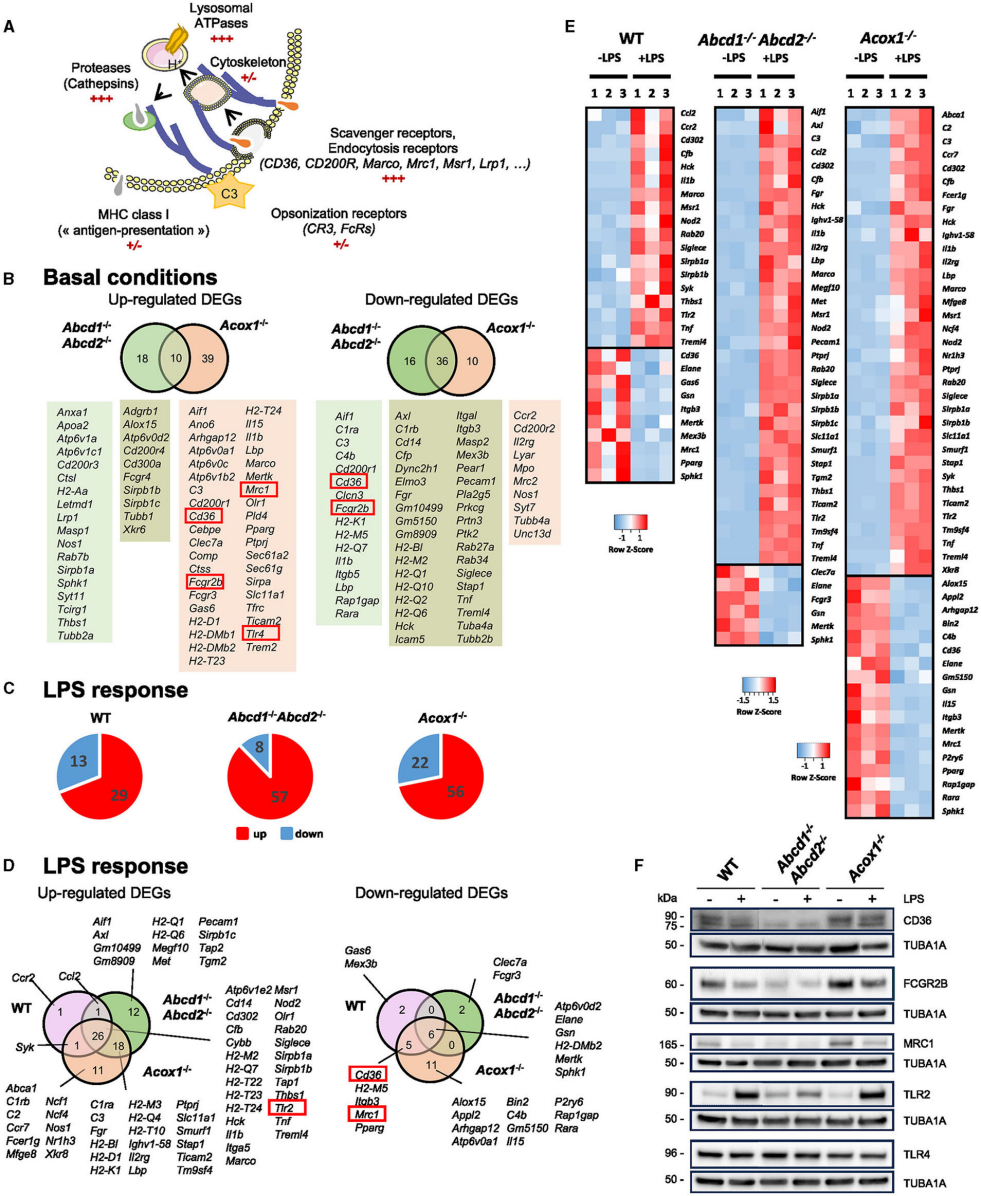
Impact of peroxisomal defect on the expression of phagocytosis-related genes in WT and mutant (*Abcd*1^−/−^*Abcd*2^−/−^ and *Acox*1^−/−^) BV-2 cells. **(A)** Schematic representation of the main actors of phagocytosis. **(B)** Venn diagrams illustrating the up- and downregulated phagocytosis-related DEGs in *Abcd*1^−/−^*Abcd*2^−/−^ and *Acox*1^−/−^ non-stimulated BV-2 cells (DataSet GSE200022). Genes whose expression level was measured at the protein level are boxed. **(C)** Pie chart displaying the number of up- or downregulated phagocytosis-related DEGs after a 24-h LPS treatment (Log2FC > 1, AdjP Value < 0.05) in *WT, Abcd*1^−/−^*Abcd*2^−/−^, and *Acox*1^−/−^ BV-2 cells (DataSet GSE237635). **(D)** Venn diagrams representing the LPS response of these DEGs (three independent batches of BV-2 cells for each genotype). **(E)** Heat map representing RNA-Seq gene expression of these DEGs at 24 h after LPS stimulation in WT, *Abcd*1^−/−^*Abcd*2^−/−^, and *Acox*1^−/−^ BV-2 cells. **(F)** Representative image of Western blotting analysis (three independent experiments) of the expression levels of CD36, FCGR2B, MRC1, TLR2, and TLR4 in WT, *Abcd*1^−/−^*Abcd*2^−/−^, and *Acox*1^−/−^ BV-2 cells untreated (–) or treated with LPS for 24 h (+). Expected molecular weights are indicated. Source data are available online for this figure.

To further define how impaired peroxisomal β-oxidation modifies other important functions of microglial cells, we first focused on the DEGs belonging to the terms “phagosome,” “Fcg Receptor,” and “engulfment.” Phagocytosis is a complex phenomenon that allows the uptake and removal of various targets (apoptotic cells and cell debris, bacteria) (Fu et al., 2014; Cockram et al., 2021). It involves many proteins of the plasma membrane serving as receptors (specific receptors for “find me” and “eat me” signals, Fc receptors, and scavenger receptors) (Figure 3A). Cytoskeleton proteins facilitate the engulfment and internalization of particles into a phagosome, while proteins in the endo-lysosomal compartment aid in the removal of degradation products, recycling, and antigen presentation (Figure 3A). In the brain, this process, mainly linked to microglia activity, participates in brain homeostasis, neural development and plasticity, synaptic connectivity, immune response, control of inflammation, and myelin repair (Galloway et al., 2019; Li et al., 2022). Several genes known to have an important role in microglial phagocytosis, especially at the cell surface, were found in the upregulated DEGs in the *Acox*1^−/−^ genotype as compared to the WT cells (*C3, Cd200r1, Cd36, Fcgr2b, Fcgr3, Lbp, Marco, Mrc1, Tlr4*, and *Trem2*) (Figure 3B). In the enrichment analysis of the *Abcd*1^−/−^*Abcd*2^−/−^ cells, we did not find the term “phagosome”. Although *Fcgr3, Tlr4*, and *Trem2* figured in the upregulated DEGs, their fold changes were below the threshold (0.78 < log2 FC < 1), and the other phagocytosis-associated genes were either not dysregulated or found in the downregulated DEGs (Figure 3B). LPS treatment resulted in a more profound change in the expression of phagocytic genes in mutant cells than in WT cells (Figures 3C, D). Some DEGs belonging to the intersection of the three genotypes were induced, such as *Msr1, Marco*, and *Tlr2*, while others were repressed, such as *Cd36, Mertk*, or *Mrc1* (Figures 3D, E). Of note, the expression levels of *Trem2* and *Tyrobp* (DAP12), which together promote microglial proliferation and phagocytosis, were not modified upon LPS treatment. Altogether, the transcriptomic data suggested different behaviors of the cells depending on the genotype, the conditions of activation, and the nature of the substrates to bind and engulf (Cockram et al., 2021).

To confirm these observations, we analyzed the expression of some of the phagocytosis markers cited above by Western blotting (Figure 3F). The lower expression of FCGR2B (Fc Gamma Receptor IIb), MRC1 (CD206), and CD36 in the *Abcd*1^−/−^*Abcd*2^−/−^ cells and their higher expression in the *Acox*1^−/−^ cells were confirmed (Figure 3F). Upon LPS treatment, we observed a weak reduction of the signal intensity for each receptor in accordance with the transcriptomic results (Figure 3F). In basal conditions, TLR4 expression did not change between genotypes, and TLR2 expression was found to only increase in the *Abcd*1^−/−^*Abcd*2^−/−^ cells. However, upon LPS treatment, we confirmed the upregulation of TLR2 with no change in the TLR4 expression. Of note, TLRs are not directly considered phagocytic receptors, but they directly modulate phagocytosis (Fu et al., 2014). A densitometric analysis of the blots is presented in Supplementary Figure 2.

**Figure 4:**
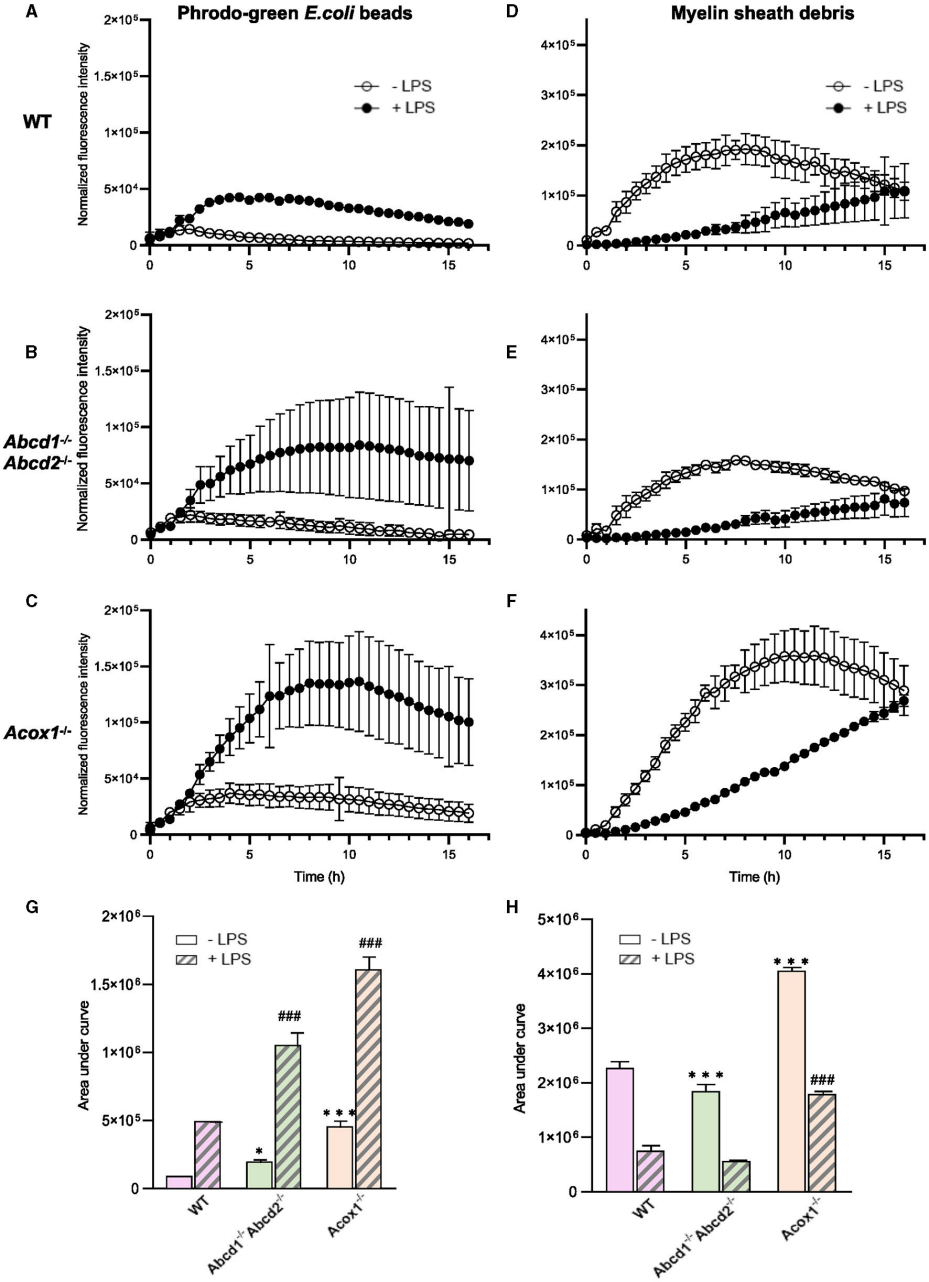
Phagocytosis of pHrodo^®^-Green *E.coli* BioParticles and pHrodo^®^-Green–labeled myelin sheath debris by BV-2 WT, *Abcd*1^−/−^*Abcd*2^−/−^, and *Acox*1^−/−^ cells. BV-2 cells were incubated with 25 μg/mL pHrodo^®^Green *E.coli* BioParticles **(A–C)** or 10 μg/mL pHrodo^®^-green–labeled myelin **(D–F)** in the presence or absence of LPS (LPS 1 μg/mL for 24 h). IncuCyte^®^ was used to monitor the phagocytosis. The time course of pHrodo^®^ signal quantification for 16 h is represented for WT **(A, D)**; *Abcd*1^−/−^*Abcd*2^−/−^ **(B, E)**; and *Acox*1^−/−^ **(C, F)** with bioparticles and myelin, respectively. The curves show the mean ± S.E.M. from *n* = 3 independent experiments for the beads **(left)** and *n* = 4 for the myelin **(right)**. Quantification of pHrodo signal over 16 h represented as area under the curve for phagocytosis of beads **(G)** and myelin **(H)**. The statistical differences were calculated using 2-way ANOVA followed by correction for false discovery rate and represented as “*” to compare the phagocytosis of KO to WT cells and as “#” to compare the LPS-treated KO vs. LPS-treated WT cells (*p < 0.05, ***p < 0.001, ^###^*p* < 0.001).

We then investigated the phagocytic capacity of WT and mutant BV-2 cells. First, we used pHrodo^®^Green *E. coli* BioParticles Conjugate, which are pH-sensitive dye conjugates that only produce bright green fluorescence when taken up into the acidic phagosome compartment. Cells were pre-incubated with LPS (1 μg/mL) for 24 h before pHrodo^®^Green *E. coli* BioParticles (25 μg/mL) were added. IncuCyte^®^ live imaging was used to monitor the engulfment (live-cell images were acquired every 30 min for 16 h, and fluorescence intensity was measured and normalized). Internalization was detectable 1 h after incubation with bioparticles (Figures 4A, B, C, G; Supplementary Figure 3). The fluorescence increased to reach a peak (2 h for the WT and *Abcd*1^−/−^*Abcd*2^−/−^ cells and 4 h for the *Acox*1^−/−^ cells) and then decreased in a time-dependent manner, possibly because of the release of the fluorochrome from the internalized particles. The mutant cells demonstrated a stronger phagocytic ability than WT cells, with *Acox*1^−/−^ cells showing the most prominent activity. In the presence of LPS, phagocytosis of fluorescent bioparticles was increased for each genotype, but the peak was delayed (5 h for the WT cells and 10 h for the mutants) (Figures 4A, B, C, G). We next evaluated the phagocytosis of YG carboxylate beads by flow cytometry 1 h after incubation with the beads (Supplementary Figure 4). While *Abcd*1^−/−^*Abcd*2^−/−^ cells demonstrated almost no phagocytosis of these beads, we were able to confirm the increased phagocytic capacity for the *Acox*1^−/−^ cells. Again, the 24 h LPS pre-treatment resulted in an increased phagocytosis capacity for each genotype. Since a main function of microglial cells is the phagocytose of myelin debris under conditions of demyelination, we next compared the phagocytic capacity of WT and mutant BV-2 cells using pHrodo^®^Green-labeled murine myelin as substrate. Accordingly, BV-2 microglial cells were incubated with pHrodo^®^Green-labeled myelin, and the uptake was followed by IncuCyte^®^ live-imaging for up to 16 h (Figures 4D, E, F, H). Internalization was detectable 4 h after incubation, increased to reach a peak (8 h for the WT and *Abcd*1^−/−^*Abcd*2^−/−^ cells and 12 h for the *Acox*1^−/−^ cells), and then decreased along with the incubation time. While the *Abcd*1^−/−^*Abcd*2^−/−^ cells showed a lower phagocytic ability than WT cells, the phagocytic rate was significantly increased in *Acox*1^−/−^ cells. Of note, the LPS treatment resulted in a decreased phagocytosis of myelin for each genotype, thus reflecting the downregulation of phagocytic receptors observed in Western blot analysis (Figure 3F). At 16 h, a continuous increase of fluorescence was observed for LPS-treated cells. In summary, peroxisomal mutations impact phagocytosis both for myelin debris and fluorescent particles with different compositions. In agreement with the transcriptomic data, the *Acox*1^−/−^ cells showed increased phagocytic properties, while *Abcd*1^−/−^*Abcd*2^−/−^ cells are less efficient than WT cells.

### Antigen presentation to T lymphocytes

**Figure 5:**
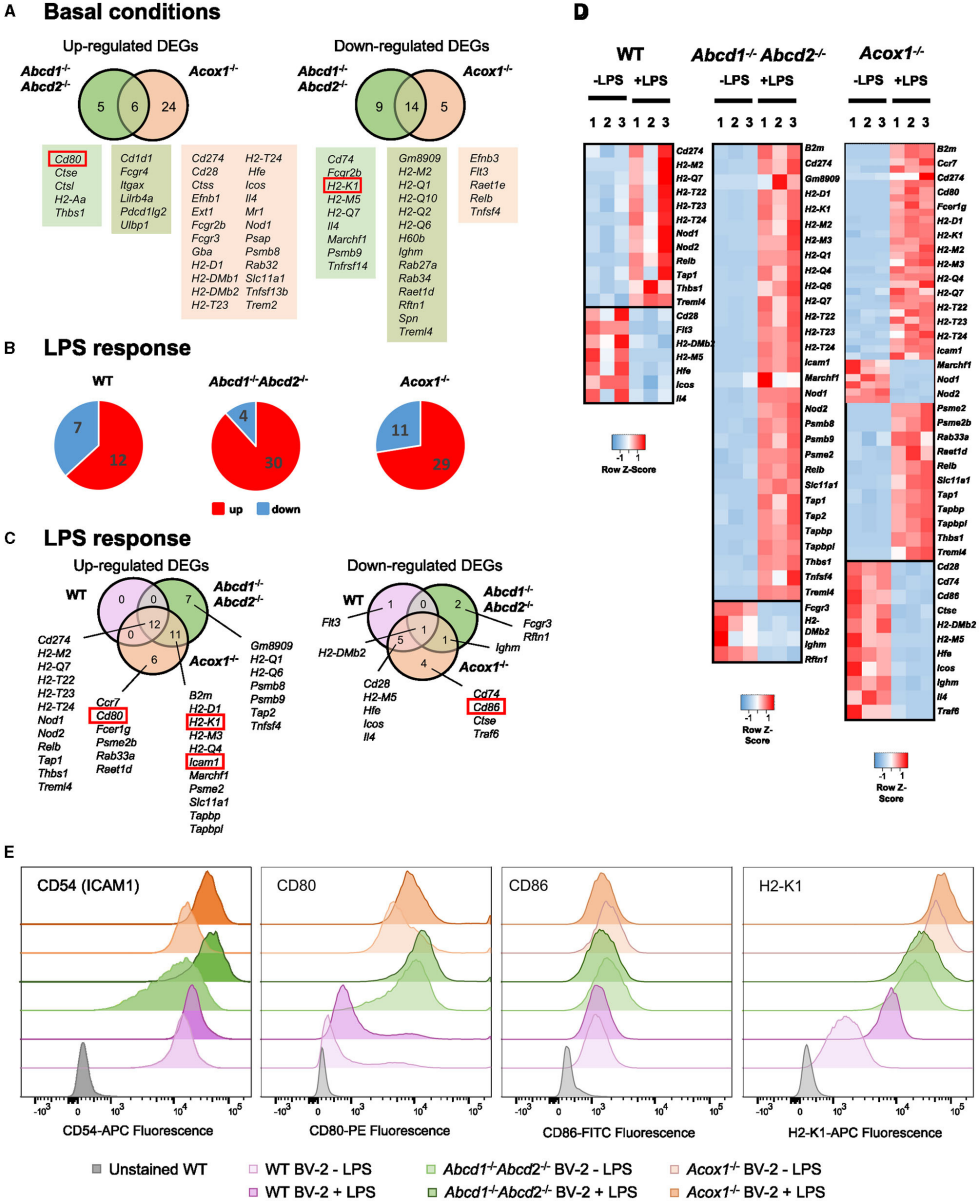
Impact of peroxisomal defect on the expression of genes related to antigen presentation and T lymphocyte co-stimulation in WT and mutant (*Abcd*1^−/−^*Abcd*2^−/−^ and *Acox*1^−/−^) BV-2 cells. **(A)** Venn diagrams illustrating the up- and downregulated DEGs (Log2FC > 1, AdjP Value < 0.05) related to antigen presentation and T lymphocyte co-stimulation in *Abcd*1^−/−^*Abcd*2^−/−^ and *Acox*1^−/−^ non-stimulated BV-2 cells (DataSet GSE200022). Genes whose expression level was measured at the protein level are boxed. **(B)** Pie chart displaying the number of these up- or downregulated DEGs after a 24-h LPS treatment (Log2FC > 1, AdjP Value < 0.05) in *WT, Abcd*1^−/−^*Abcd*2^−/−^, and *Acox*1^−/−^ BV-2 cells (DataSet GSE237635). **(C)** Venn diagrams representing the LPS response in each genotype of these DEGs (three independent batches of BV-2 cells for each genotype). **(D)** Heat map representing RNA-Seq gene expression (with Z-scores) of these DEGs at 24 h after LPS stimulation in WT, *Abcd*1^−/−^*Abcd*2^−/−^, and *Acox*1^−/−^ BV-2 cells (three independent batches of BV-2 cells for each genotype). **(E)** Flow cytometry analysis of the expression level of CD54 (ICAM1), CD80, CD86, and MHC class I protein H2-K1 in WT, *Abcd*1^−/−^*Abcd*2^−/−^, and *Acox*1^−/−^ BV-2 cells surface stained with fluorescent-labeled monoclonal antibodies (FITC, Fluorescein isothiocyanate; PE, phycoerythrin; APC, Allophycocyanin). The results are plotted as a distribution histogram of fluorescence intensities (arbitrary units) of populations of living BV-2 cells. Unstained BV-2 cells were shown in gray to evaluate intrinsic autofluorescence, compared to fluorescent antibody-stained cells (WT BV-2 ± LPS in light/dark purple, *Abcd*1^−/−^*Abcd*2^−/−^ ± LPS in light/dark green, and *Acox*1^−/−^ ± LPS in light/dark orange, respectively). The results are representative of four independent experiments.

Microglia plays a crucial role in the immune response as the primary antigen-presenting cell (APC) in the brain parenchyma, particularly during neurodegeneration. They express both major histocompatibility complex (MHC) class I and class II molecules, enabling them to activate infiltrating CD8^+^ and CD4^+^ T lymphocytes, respectively (Schetters et al., 2017). Our transcriptomic analysis revealed a significant number of DEGs among the genes involved in antigen processing and presentation, particularly in the *Acox1*^−/−^ genotype (Figure 5A). Many of these genes were H2 genes, which encode MHC proteins (Figure 5A). The response to LPS was more pronounced in the mutant cell lines compared to the WT cells (Figures 5B, C). As expected, LPS treatment primarily induced the expression of genes involved in antigen processing, such as *Tap1* and antigen presentation (H2 genes). Interestingly, MHC class I genes *H2-D1* and *H2-K1* were only induced in the mutant cells along with *B2m*, suggesting a skewing toward CD8^+^ T lymphocyte activation (Figures 5C, D). In addition, genes involved in adhesion [*Icam1* (*Cd54*)] and co-stimulation (*Cd80*) were also upregulated only in the mutant genotypes (Figures 5C, D). Of note, the expression of *Gpnmb* and *Lilrb4* genes, which encodes for reliable markers of microglial activation, considered as anti-inflammatory and immunosuppressive modulators of adaptative immunity (Kretzschmar et al., 2021; Saade et al., 2021), has been found induced in BV-2 mutant cells (Raas et al., 2023). Moreover, we observed modifications of the expression of several genes involved in the suppression of microglia and APC-mediated T cell activation [induction of *Trem*2, *Tyrobp* (DAP12), *CD200r3, CD200r4*, and repression of *Cx3cr1* and *Itgam* (CR3), while *Cd200r1* was repressed in *Abcd1*^−/−^*Abcd2*^−/−^ cells and induced in *Acox1*^−/−^ cells]. Altogether, these data suggest that peroxisomal defects alter the immune response and modify antigen presentation to T lymphocytes.

**Figure 6:**
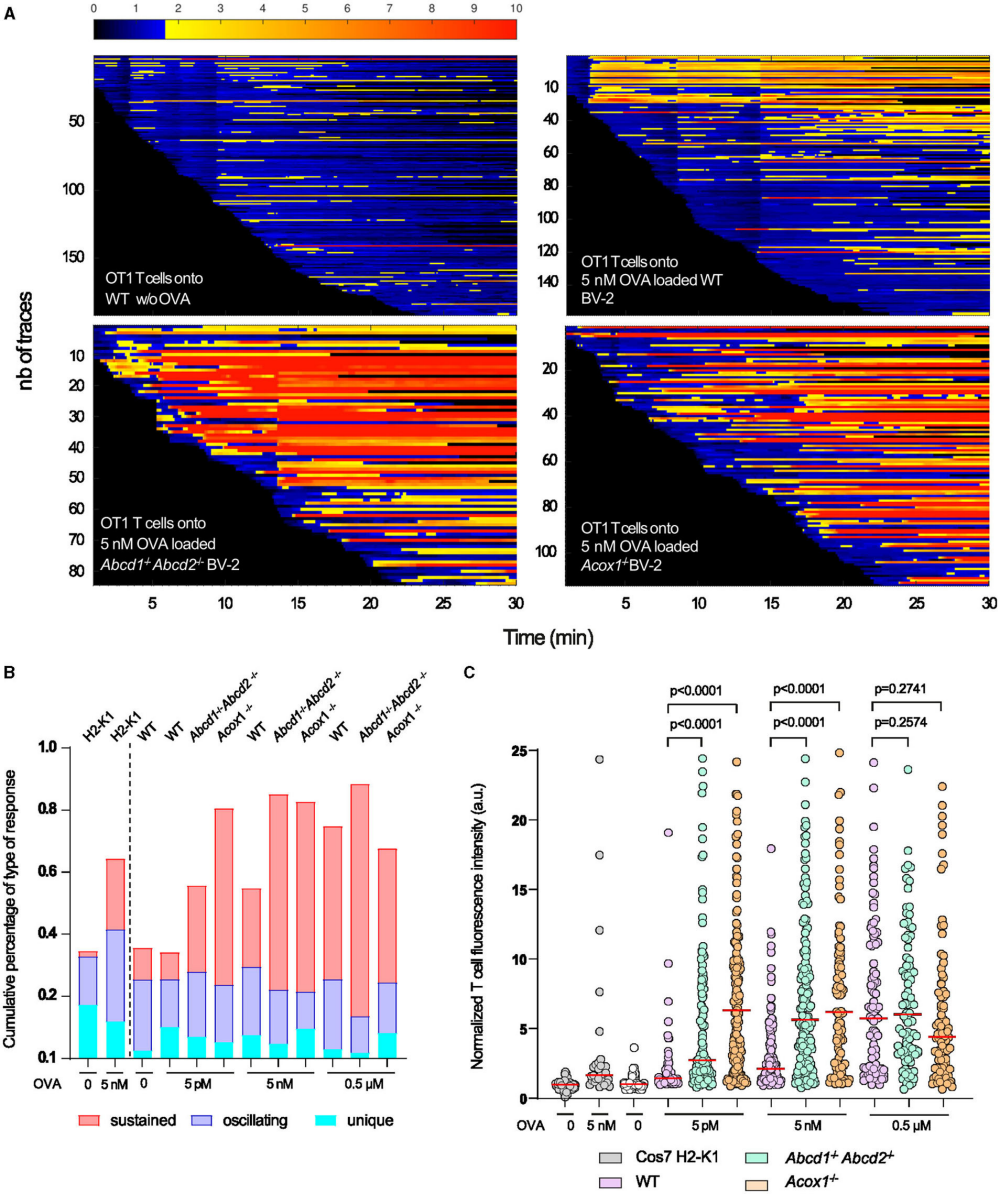
T cell calcium fluctuations as a consequence of BV-2 antigen presentation. OT1 CD8+ T cells were loaded with the PBX fluorescent calcium indicator prior to being seeded onto WT, *Abcd*1^−/−^*Abcd*2^−/−^, and *Acox*1^−/−^ BV-2 cells previously treated overnight with LPS and pulsed with various quantities of agonistic OVA-derived peptide (N4-SIINFELKL) and acting as antigen-presenting cells. The amplitude of the calcium response was quantified by MAAACS. **(A)** Overall representation as a barcode of each tracked T cell ordered as a function of their early detection. Normalized fluorescence intensity is color-coded with the above look-up table, where the activation threshold is set to 1.7 a.u. (blue to yellow transition). **(B)** Upon the MAAACS analysis, the global overview of the cell response heterogeneity is summarized in a cumulative histogram where the percentage of cells are plotted displaying a unique calcium response (only 1 peak above threshold), oscillating (fluorescence oscillations above threshold representing between 20 and 60% of the total trace) and sustained (more than 60% of the recorded normalized fluorescence above threshold). **(C)** Dot plot representation of the normalized fluorescence intensity of each T cell above threshold in various experimental conditions (increasing quantities of agonistic OVA derived peptide). As a control, Cos7 cells expressing H2-K1 MHCI molecules devoid of costimulatory molecules were used to trigger MHC-TcR restricted calcium response in OT1 CD8^+^ T cells. Statistical significance was calculated according to the Mann–Whitney test.

To address this hypothesis, we first analyzed by flow cytometry the expression of some cell surface proteins involved in antigen presentation H2-K1 (H2-Kb), co-stimulation (CD80 and CD86), and adhesion CD54 (ICAM1). In basal conditions, we confirmed the upregulation of CD80 in both mutant cells, while H2-K1 expression was paradoxically increased in the mutant cells (Figure 5E). Upon LPS treatment, the results confirmed a higher expression of H2-K1, CD80, and ICAM1 in the mutant genotypes and a downregulation of CD86, and its expression level remained equivalent to that found in WT cells (Figure 5E). To functionally link these expression levels to T cell activation, we performed calcium response imaging in ex-vivo naïve CD8+ OT1 T cells by live imaging. Calcium fluctuations were further analyzed using our home-made algorithm MAAACS (methods for automated and accurate analysis of cell signals) (Salles et al., 2013; Sadoun et al., 2021) of the calcium flux in T lymphocytes upon stimulation by OVA-loaded LPS-treated BV-2 cells acting as antigen-presenting cells (Figure 6). We mainly analyzed the calcium fluctuations, the intensity, and the duration of the response over the threshold. This enables to classify responses as unique, oscillating, or sustained according to the duration of the calcium increase over the threshold and the frequency of calcium peaks. Throughout a 1-h timelapse, 35–90% of T cells displayed a calcium response over the threshold but were mostly dependent (in terms of intensity) when T cells were seeded onto antigen-loaded BV-2 cells. We evidenced that the mutant BV-2 cells, compared to WT BV-2, were inducing a strong calcium response in T cells, dominantly sustained even for low doses of antigen (in the range of a few pM). This may originate from the enhanced surface expression of adhesion and co-stimulatory molecules that drives T cell response upon a sustained calcium response by the co-engaging of the TCR, CD28, and LFA-1, consistent with our previous observations (Xia et al., 2018). Indeed, when CD8^+^ OT1 cells were seeded onto COS7 APC expressing only MHC1 molecules, both the frequency of activated T cells, the strength, and the shape of the response (sustained vs. oscillating) were far less intense in T cells than those obtained in contact with mutant BV-2 cells (Figure 6).

## Discussion

In this study, we further characterized mutant microglial BV-2 cells with peroxisomal defects by focusing on the immune response and investigating their response to LPS stimulation. Our aim was to better understand the impact of peroxisomal β-oxidation on microglial functions and to help clarify the pathophysiology of X-ALD and other peroxisomal leukodystrophies. Microglia play an essential role in mediating the immune response in the brain through their ability to detect danger, participate in the elimination of damaged or dead cells, trigger, amplify, and resolve inflammation, present antigens and induce the lymphocyte response, and control the blood–brain barrier. Inflammation is not always harmful but rather an adaptive response to damage that can sometimes become unmanageable (Sochocka et al., 2017). Neuroinflammation occurs when microglia become overactive and release inflammatory cytokines and reactive oxygen species, leading to infiltration of peripheral immune cells and the destruction of healthy neurons and synapses. The abnormal activation of microglia can lead to devastating outcomes, including neurodegenerative diseases. Therefore, understanding the mechanisms of microglial activation and how to regulate their activity is crucial for developing therapies for neurodegenerative diseases. We previously demonstrated that the *Abcd*1^−/−^*Abcd*2^−/−^ and *Acox*1^−/−^ cells have lost their homeostatic state (Raas et al., 2023). Concerning inflammation, in basal conditions, in spite of the upregulation of *Trem2* and the presence of a DAM-like signature in mutant BV-2 cells, pro-inflammatory genes were mostly repressed (Raas et al., 2023). A clear polarization toward an anti-inflammatory state was, however, absent in these conditions.

LPS-stimulated BV-2 microglial cells are often used as *in vitro* models of neuroinflammation, and accordingly, BV-2 cells adopt a classical pro-inflammatory state (induction of *Il1b, Il6*, and *Tnf* ). In our study, LPS treatment induced an inflammatory response in both WT and mutant BV-2 cells, illustrated by the upregulation of NLRP3 and the increased secretion of several pro-inflammatory cytokines and chemokines. It is important to note that our results recapitulate most of the results already published on WT BV-2 cells but minor discrepancies exist and probably result from the use of different LPS types along with different concentrations (from 10 ng/mL to 1 μg/mL) and lengths of treatment (from 4 to 24 h) (Henn et al., 2009; Dai et al., 2015; Das et al., 2015, 2016; Bussi et al., 2017). Of note, we observed that the overall LPS response was largely increased in the mutant genotypes, both in the number of DEGs and in their range of variation. Such amplified response has already been described in primed microglia, microglia of the aged brain (Norden and Godbout, 2013; Perry and Holmes, 2014; Haley et al., 2019). This finding suggests that peroxisomal defects could serve as a potential trigger, contributing to the dysfunction of microglia during the aging process. Pathogen recognition receptors, including TLR4, which mediates inflammatory responses by recognizing lipopolysaccharide (LPS) and TLR2 (Lehnardt et al., 2002; Hwang et al., 2016), could be directly involved in this amplified response since their expression is increased in the mutant cell lines, in basal condition for *Tlr4*, and upon LPS stimulation for *Tlr2*. Of note, other TLR encoding genes figured among the DEGs both in basal conditions (induction of *Tlr1, Tlr3*, and *Tlr13* in the mutant cells) and after LPS treatment (induction of *Tlr3* and *Tlr12* and repression of *Tlr5* and *Tlr8*), suggesting that peroxisome defect impacts not only neuroinflammation but also adaptive immunity and antiviral response (Kumar, 2019; Dabi et al., 2023).

Confirmation of the induction at the protein level was obtained from selected proteins (CCL2, CCL3, CCL4, and TNF). The increased LPS-dependent secretion of TNF, which is considered a major cytokine in neurodegenerative processes (Montgomery and Bowers, 2012), was significantly higher in mutant cells than in WT cells consistently with the LPS response of peripheral blood mononuclear cells of X-ALD patients (Lannuzel et al., 1998; Di Biase et al., 2001). The *Tnf* gene was investigated as a possible modifying gene related to the different clinical phenotypes of X-ALD, but the conclusions rule out this hypothesis (McGuinness et al., 1995). More recent analyses failed to demonstrate the correlation between plasma levels of TNF and the severity of the disease (Marchetti et al., 2018). Regarding IL1B, we failed to detect secretion of this cytokine both in mutant and WT BV-2 cells, which is in contradiction with previous studies for the WT cells (Dai et al., 2015; Bussi et al., 2017). Our results are consistent with the lack of maturation of CASP1 and IL1B, which is likely attributed to the significantly low expression level of *Asc* in our cell lines. Nevertheless, we observed an enhanced expression of *Ilb* mRNA and pro-IL1B protein in all genotypes following LPS treatment. This finding aligns with the enrichment of NF*-*κB signaling in the upregulated DEGs. Notably, in basal conditions, we did not observe a significant upregulation in mRNA of *Il1b* when compared to WT contrary to what was seen in *Abcd*1^−/−^ mice (Gong et al., 2017). As expected from previous studies (Lu et al., 2013), the CCL2, CCL3, and CCL4 chemokines showed increased secretion levels upon LPS treatment. Noteworthy, the highest concentrations were obtained in the mutant supernatants. These chemokines are small cytokines that play a key role in the regulation of immune cell migration and actively participate in neurodegenerative processes (Wojcieszak et al., 2022). CCL2 (MCP-1) is the ligand of the CCR2 receptor and is involved in the recruitment of peripheral immune cells to sites of inflammation. CCL3 (MIP-1α) binds CCR5 and has a similar function as CCL2 but has also been reported to mediate immune cells' cytokine secretion and promote aggregation and migration of various cells. CCL4 (MIP-1β), another ligand of CCR5, is implicated in the attraction of natural killer cells, monocytes, and some other inflammatory cells. *Tnf* , *Ccl2*, and *Ccl4* mRNA have been shown to be increased in the inflammatory areas of the brain affected by cALD (Paintlia et al., 2003). Cerebrospinal fluid revealed higher levels in cALD samples compared to controls for CCL2 and CCL3, and serum CCL2 significantly correlated with disease severity as determined by MRI (Lund et al., 2012). Altogether, our results suggest that peroxisomal defect in BV-2 microglial cells is responsible for an inflammatory over response when stimulated by LPS and that the excessive release of pro-inflammatory cytokines and chemokines by microglia would be a major cause of neurotoxicity. Despite the involvement of other cell types (mainly astrocytes or infiltrating immune cells) in the release of these cytokines within the brain, we can, therefore, speculate that neuroinflammation may arise due to an excessive reaction of microglia. In X-ALD patients, such a hyper-response would be triggered by internal or external stimuli and amplified by the progressive accumulation of VLCFA. Such a hypothesis is in agreement with a recent study in human macrophages showing that ABCD1 defect and VLCFA accumulation increase their proinflammatory response (Zierfuss et al., 2022).

In this study, we further explored phagocytosis, another important function of microglia. In physiologic situations, phagocytosis is critical for maintaining brain homeostasis, participating in synaptic plasticity, and the continuous removal of debris and dying cells (Galloway et al., 2019; Hammel et al., 2022). Clearance of dying cells and debris allows the recruitment of oligodendrocyte progenitor cells and remyelination (Lloyd and Miron, 2019). Moreover, microglial phagocytosis of myelin was shown to be tightly linked to a decreased inflammatory response (Liu et al., 2006). Conversely, in the disease state, phagocytosis alteration largely contributes to neuroinflammation and demyelination and, thus, constitutes a therapeutic target in neurodegenerative disorders. Microglial autophagy-associated phagocytosis is responsible for myelin degradation and is essential for recovery from neuroinflammation (Berglund et al., 2020).

Here, we showed that peroxisomal defects in BV-2 cells impact the phagocytosis of myelin and *E. coli* BioParticles or carboxylate beads in different ways depending on the nature of the substrates. Carboxylate beads were almost not engulfed by *Abcd*1^−/−^*Abcd*2^−/−^ cells, while *Acox*1^−/−^ cells displayed a 2-fold increased ability as compared to WT. *Abcd*1^−/−^*Abcd*2^−/−^ cells engulfed bioparticles (pHrodo^®^Green *E. coli* BioParticle Conjugate) slightly more efficiently than WT cells but were less potent than *Acox*1^−/−^ cells. Independently of genotype and the type of particles, LPS treatment resulted in an increased phagocytosis ability. Regarding phagocytosis of labeled myelin debris, while *Abcd*1^−/−^*Abcd*2^−/−^ cells were slightly less potent than WT cells, *Acox*1^−/−^ cells again displayed much more efficiency. Unlike artificial beads, the LPS treatment resulted in a decreased phagocytic ability of myelin debris independent of the genotype. Surprisingly, knockouts of peroxisomal ABC transporters and ACOX1 enzyme gave different results, which could be explained by the differential expression of several key genes encoding various receptors involved in phagocytosis such as CD36, which was found to be associated with the uptake of myelin debris (Grajchen et al., 2020) or complement-receptor-3 (CR3) (van der Laan et al., 1996; Reichert and Rotshenker, 2003; Zorina et al., 2018), low-density lipoprotein receptor-related protein 1 (LRP1) (Gaultier et al., 2009), Fc-receptors (Kuhlmann et al., 2002), MerTK (Healy et al., 2017; Shen et al., 2021), macrophage scavenger receptor 1 (MSR1, CD204) (Kong et al., 2020), scavenger receptors [SR-AI/II and collectin placenta 1 (CL-P1)] (Reichert and Rotshenker, 2003), and TREM2 (Cignarella et al., 2020). Microglia expressing high levels of mannose receptor MRC1 (CD206) are abundant in active lesions, and this receptor also promotes remyelination (Lloyd and Miron, 2019). Most of the genes encoding these receptors were found upregulated only in the *Acox*1^−/−^ genotype. In addition, *Cd36* and *Fcgr2b* were downregulated in the *Abcd*1^−/−^*Abcd*2^−/−^ cells. Regarding the different results obtained with LPS, we can point out the downregulation of *Mertk* in each genotype and *Cd36* and *Mrc1* in WT and *Acox*1^−/−^. Upon LPS treatment, the decreased expression of *Cd36*, which was shown to promote myelin phagocytosis (Grajchen et al., 2020), and *MertK*, known as a positive regulator of myelin phagocytosis (Healy et al., 2017), are in agreement with the reduced phagocytosis of myelin debris observed in BV-2 cells. Regarding fluorescent bioparticles and beads, LPS treatment resulted in increased phagocytosis, suggesting that the bioparticles and beads we used do not involve the same receptors involved in myelin phagocytosis. TLR2 and MARCO, whose expression was increased by LPS, could be one of the surface proteins involved in the increased phagocytosis of coated beads. The use of blocking antibodies in another phagocytosis assay with BV-2 cells and phycoerythrin-conjugated polystyrene microspheres demonstrated their involvement (Rangaraju et al., 2018).

In the context of X-ALD, changes in the process of phagocytosis in the brain could serve as a crucial event triggering the development of demyelination and neurodegeneration and impacting myelin repair (Gong et al., 2017; Bergner et al., 2019, 2021). Phagocytosis indeed permit to eliminate damaged myelin, to resolve inflammatory signals, and to scavenge the lipotoxic effects of VLCFA in the other brain cell. However, we can speculate on the negative outcomes associated with both an increase and a decrease in the ability to undergo phagocytosis. Increased phagocytosis of myelin debris containing VLCFA could form foamy microglia, which, once surpassing a certain threshold, could become fully dysfunctional, particularly in terms of their role in regulating inflammation. Conversely, a reduced phagocytosis capability would hinder the elimination of accumulated VLCFA and abnormal myelin, thus promoting cell death. In our study, contrary to the *Acox*1^−/−^ genotype, *Abcd*1^−/−^*Abcd*2^−/−^ cells displayed a decreased ability to myelin phagocytosis. *Abcd*1^−/−^ mouse primary microglia, in both unstimulated and LPS-challenged conditions, demonstrated upregulation of phagocytosis-related genes, including *Trem*2 but not *C3* (Gong et al., 2017). Phagocytosis of labeled neurons exposing phosphatidylserine was weakly increased in the absence or presence of LPS (Gong et al., 2017).

The defective interplay between microglia and T lymphocytes is now recognized as essential in various neurodegenerative diseases, including Alzheimer's disease (AD) and Parkinson's disease (PD) (Schetters et al., 2017; Dressman and Elyaman, 2022; Xu et al., 2023). Microglia and T lymphocytes are indeed tightly linked in terms of activation and function. Many microglial-secreted cytokines and chemokines are known to recruit and control T lymphocyte activity. Microglia also play a major role in the priming of T lymphocyte response through their ability to present antigens (Aloisi et al., 1999). Several alleles of HLA genes, which code for components of MHC class I or class II expressed by microglia, are now considered risk factors in these diseases (Xu et al., 2023). Here, we demonstrated that a defect in peroxisomal β-oxidation in BV-2 cells modifies the expression of numerous genes involved in this cellular crosstalk between microglia and T lymphocytes. Upon LPS activation, the expression of key markers of antigen presentation and co-stimulation were also modified. More importantly, we showed for the first time that peroxisomal mutations in microglia, acting as APCs, have a significant impact on the T lymphocyte response. This effect likely stems from alterations in the expression of MHC, adhesion, and co-stimulatory surface molecules, especially upon LPS activation. In X-ALD, accumulation of T lymphocytes has been found in brain lesions (Griffin et al., 1985) and likely results from the microglial-dependent rupture of the blood–brain barrier and the secreted chemoattractants. Together with infiltrating macrophages, T lymphocytes form a substantial portion of inflammatory cells following the demyelination's forefront. This observation implies that infiltration and inflammatory activation are subsequent events that follow demyelination (Eichler and Van Haren, 2007). Over-reactive microglia would permit to initiate and amplify such a situation. Our results reinforce the importance of microglia interplay with T lymphocytes, possibly participating in a loop of amplification leading to chronic inflammation and neurodegeneration. In the context of X-ALD, targeting microglia and their capacity to prime T lymphocytes makes sense from a therapeutic point of view, as it was proven to be efficient in a mouse model of Alzheimer's disease (Chen et al., 2023). However, the lack of predominant T-cell Vbeta receptor usage in CALD patients effectively ruled out the hypothesis, suggesting a link between T-cell repertoire and clinical variability in X-ALD (Picard et al., 2005).

While the alterations in the expression levels of various surface proteins related to antigen presentation, T cell co-stimulation, and phagocytosis provide a plausible explanation for the observed outcomes, we cannot exclude that lipid modifications may change the biophysical properties of the plasma membrane and its lateral and transversal organization, affecting the functionality of the surface receptors as evidenced for the interferon-gamma receptor, CD28, and the PI3K/AKT axis (Lasserre et al., 2008; Blouin et al., 2016). Both phagocytosis (Nadjar, 2018; Rubio et al., 2018) and T cell activation are indeed impacted by such modifications (Chouaki Benmansour et al., 2018). We have previously demonstrated a vast reprograming of lipid metabolism and changes in the fatty acid composition in the mutant cell lines, together with the accumulation of cholesterol, especially in the plasma membrane (Raas et al., 2019a,b, 2023). Additional research is required to analyze the biophysical properties of the plasma membrane in BV-2 microglial cells and investigate how these putative peroxisomal-dependent alterations on membrane reorganization may affect phagocytosis and cell-to-cell interactions.

Taken together, our study reveals that impaired peroxisomal β-oxidation transforms murine BV-2 microglial cells into over-reactive microglia in which the secretion of inflammatory cytokines, the phagocytosis ability, and the antigen presentation to T lymphocytes are drastically modified, especially upon stimulation. This finding further highlights the pivotal role of immunometabolism in the context of X-ALD and peroxisomal leukodystrophies, shedding light on the pathophysiological mechanisms underlying these disorders. We acknowledge that further studies using primary microglia would be a valuable addition to confirm the relevance of our findings. Nevertheless, we believe that these insights might hold the potential for the development of novel therapeutic approaches targeting microglia to prevent or slow down the neuroinflammatory process in peroxisomal disorders.

## Data availability statement

The datasets presented in this study can be found in online repositories. The names of the repository/repositories and accession number(s) can be found below: https://www.ncbi.nlm.nih.gov/geo/, GSE200022; https://www.ncbi.nlm.nih.gov/geo/, GSE237635.

## Author contributions

AT: Data curation, Investigation, Writing – original draft. QR: Data curation, Investigation, Writing – original draft. MT-J: Investigation, Writing – original draft. CK: Data curation, Formal analysis, Investigation, Writing – original draft. RK: Data curation, Formal analysis, Investigation, Writing – original draft. DT: Investigation, Writing – original draft. BN: Supervision, Writing – original draft. EB: Investigation, Writing – original draft. MD: Investigation, Validation, Writing – original draft. YH: Conceptualization, Formal analysis, Investigation, Methodology, Writing – original draft. AB: Writing – original draft. FD: Writing – original draft. TC: Investigation, Writing – original draft. JB: Writing – original draft. IW: Investigation, Writing – original draft. SM: Writing – original draft. MC-M: Data curation, Formal analysis, Investigation, Writing – original draft. PA: Data curation, Formal analysis, Investigation, Visualization, Writing – original draft. CG: Conceptualization, Data curation, Formal analysis, Investigation, Methodology, Supervision, Visualization, Writing – original draft. SS: Conceptualization, Data curation, Formal analysis, Funding acquisition, Investigation, Methodology, Supervision, Validation, Visualization, Writing – original draft.

## Supplementary material

Supplementary Figure 1 Densitometric analysis of the blots presented in Figure 2F related to cytokines and inflammation. Histograms represent the mean of the relative intensity of the signal Pro-CASP1, Pro-IL1B, NLRP3 relative to the signal corresponding to the housekeeping genes (ACTB or TUB1A1) obtained from 3 independent experiments. Results are expressed relative to the untreated WT cells. The statistical differences were calculated using 2-way ANOVA and represented as “*” to compare LPS vs. untreated cells and calculated using 1-way ANOVA and represented as '#' to compare the LPS effect on KO vs. LPS effect on WT cells (^*^*p* < 0.05, ^***^*p* < 0.001,^#^*p* < 0.05).

Supplementary Figure 2 Densitometric analysis of the blots presented in Figure 3F. Histograms represent the mean of the relative intensity of the signal CD36, FCGR2B, MRC1, TLR2, and TLR4 relative to the signal corresponding to the housekeeping genes (TUB1A1) obtained from 3 independent experiments. Results are expressed relative to the untreated WT cells. The statistical differences were calculated using 2-way ANOVA and represented as “*” to compare LPS vs. untreated cells and calculated using 1-way ANOVA and represented as ‘#' to compare the LPS effect on KO vs. LPS effect on WT cells (^*^*p* < 0.05, ^***^*p* < 0.001,^#^*p* < 0.05).

Supplementary Figure 3 Phagocytosis of pHrodo-green *E.coli* bioparticles by WT, Abcd1-/-Abcd2-/-, and Acox1-/- BV-2 cells. BV-2 cells were incubated with 25 μg/mL pHrodo-green *E.coli* bioparticles. Fluorescence images at t0 and t16 were extracted from the IncuCyte^®^ live cell imaging used to quantify florescence signal.

Supplementary Figure 4 Phagocytosis of YG beads by BV-2 cells. Following treatment (+) or not (-) with LPS 1 μg/mL for 24 h, the ability of BV-2 cells (WT, Abcd1-/-Abcd2-/-, and Acox1-/-) to phagocytose fluorescent YG carboxylate beads were performed at 37°C as indicated. **(A)** Percentage of YG-beads positive cells were determined by flow cytometry and shown as mean ± SEM, with *n* = 4 separate experiments for wild type cells and *n* = 5 separate experiments for KO cells. (B) Phagocytic index were calculated [(% YG beads-positive cells) x (mean fluorescence intensity of YG beads-positive cells)] and shown as mean ± SD, with n = 4 separate experiments for wild type cells and n = 5 separate experiments for KO cells. Statistical significance was calculated using 2-way ANOVA followed by correction for false discovery rate and represented as '*' to compare the phagocytosis of -LPS KO to WT cells and as '#' to compare the LPS-treated KO vs. LPS-treated WT cells (^*^*p* < 0.05, ^**^*p* < 0.01,^#^*p* < 0.05, ###*p* < 0.001). Negative controls for which phagocytosis was blocked by cytochalasin D treatment and by keeping cells on ice were performed with no positive cells obtained (data not shown here).

DATA SOURCE Uncropped blot for Western blot analysis of Caspase 1 (slide no. 2), Interleukin 1 beta (slide no. 3), NLRP3 (slide no. 4), CD36 (slides no. 5-6), FCGR2B slides no. 7), MRC1 (slide no. 8), TLR2 (slides no. 9), TLR4 (slide no. 10).
