# Supplementary figures and images for: Immune response of BV-2 microglial cells is impacted by peroxisomal beta-oxidation
Source: Front Mol Neurosci. 2023 Dec 18;16:1299314. doi: 10.3389/fnmol.2023.1299314 (PMC10757945; doi:10.3389/fnmol.2023.1299314)

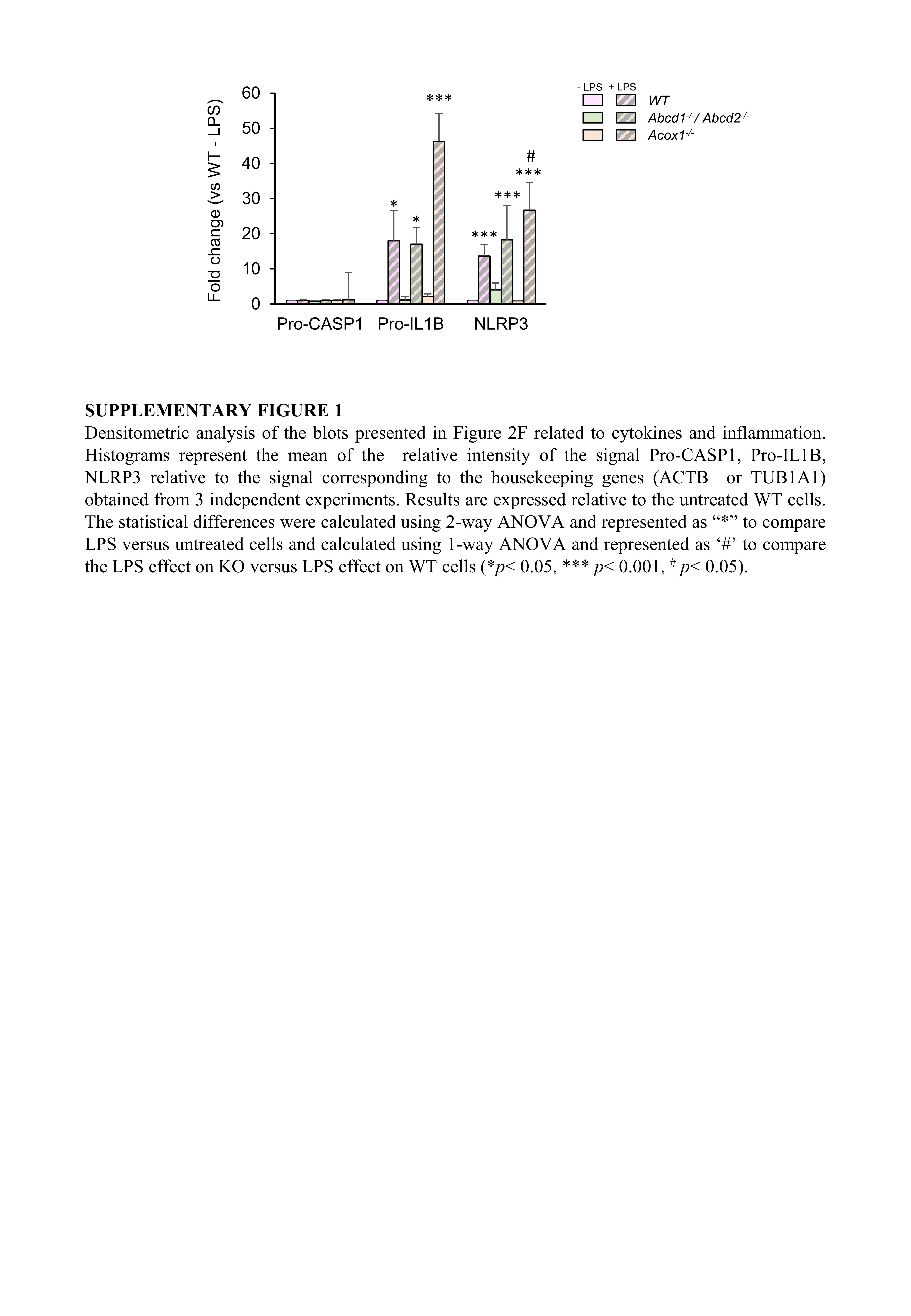

Supplement: Supplementary Figure 1 — Densitometric analysis of the blots presented in Figure 2F related to cytokines and inflammation. Histograms represent the mean of the relative intensity of the signal Pro-CASP1, Pro-IL1B, NLRP3 relative to the signal corresponding to the housekeeping genes (ACTB or TUB1A1) obtained from 3 independent experiments. Results are expressed relative to the untreated WT cells. The statistical differences were calculated using 2-way ANOVA and represented as “*” to compare LPS vs. untreated cells and calculated using 1-way ANOVA and represented as '#' to compare the LPS effect on KO vs. LPS effect on WT cells (*p < 0.05, ***p < 0.001,#p < 0.05). [file Image_1.tif]

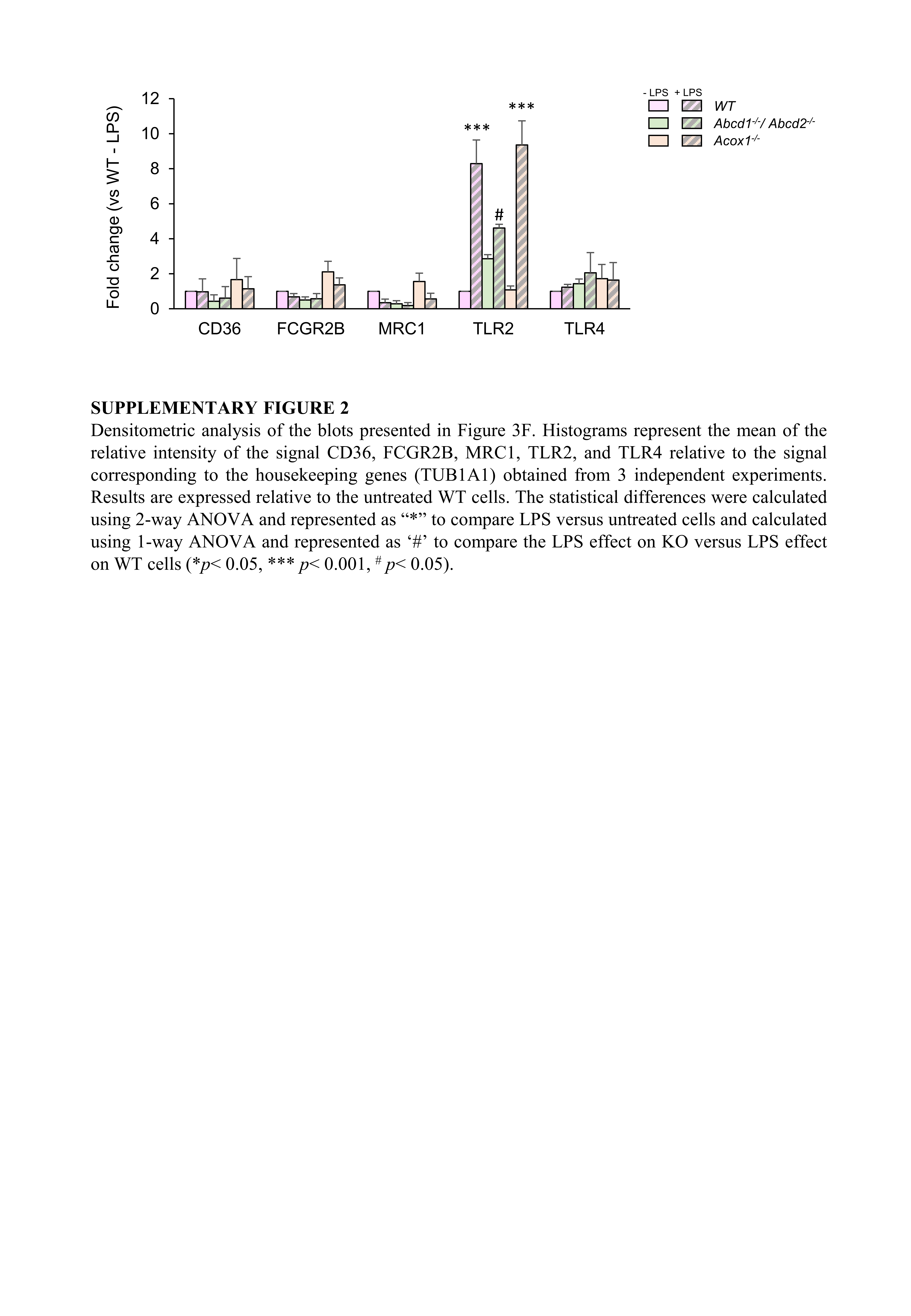

Supplement: Supplementary Figure 2 — Densitometric analysis of the blots presented in Figure 3F. Histograms represent the mean of the relative intensity of the signal CD36, FCGR2B, MRC1, TLR2, and TLR4 relative to the signal corresponding to the housekeeping genes (TUB1A1) obtained from 3 independent experiments. Results are expressed relative to the untreated WT cells. The statistical differences were calculated using 2-way ANOVA and represented as “*” to compare LPS vs. untreated cells and calculated using 1-way ANOVA and represented as ‘#' to compare the LPS effect on KO vs. LPS effect on WT cells (*p < 0.05, ***p < 0.001,#p < 0.05). [file Image_2.tif]

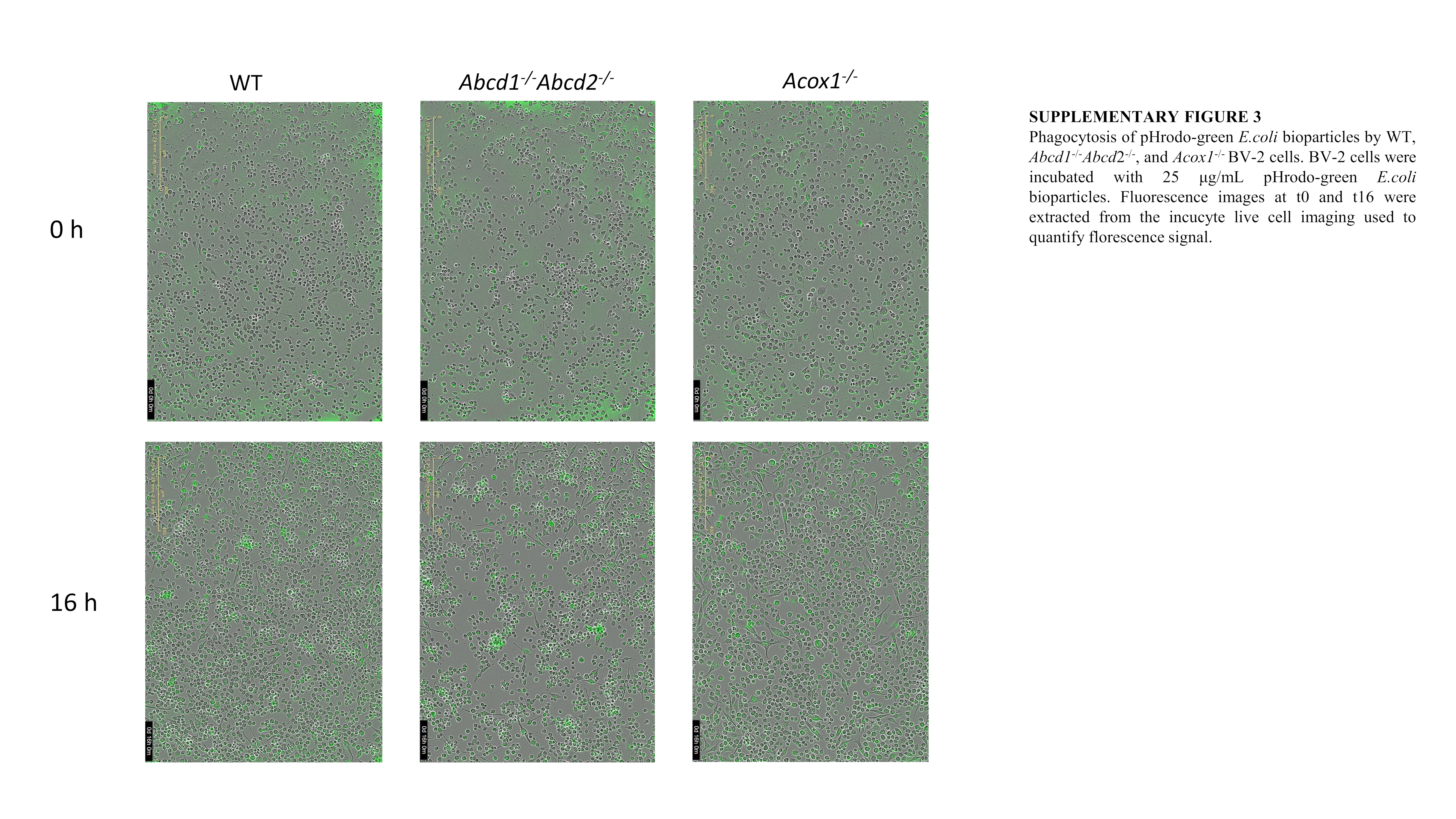

Supplement: Supplementary Figure 3 — Phagocytosis of pHrodo-green E.coli bioparticles by WT, Abcd1-/-Abcd2-/-, and Acox1-/- BV-2 cells. BV-2 cells were incubated with 25 μg/mL pHrodo-green E.coli bioparticles. Fluorescence images at t0 and t16 were extracted from the IncuCyte® live cell imaging used to quantify florescence signal. [file Image_3.tif]

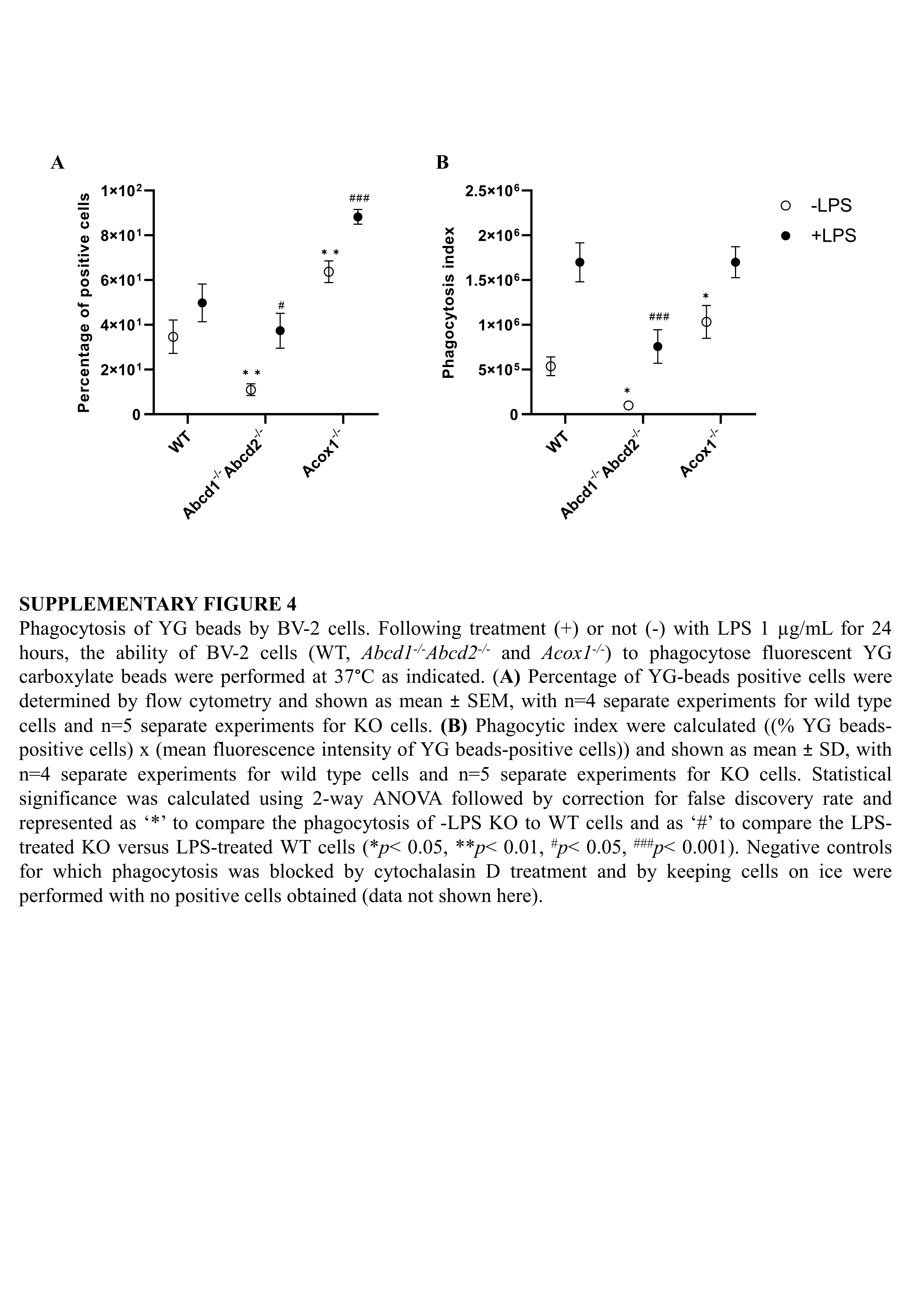

Supplement: Supplementary Figure 4 — Phagocytosis of YG beads by BV-2 cells. Following treatment (+) or not (-) with LPS 1 μg/mL for 24 h, the ability of BV-2 cells (WT, Abcd1-/-Abcd2-/-, and Acox1-/-) to phagocytose fluorescent YG carboxylate beads were performed at 37°C as indicated. (A) Percentage of YG-beads positive cells were determined by flow cytometry and shown as mean ± SEM, with n = 4 separate experiments for wild type cells and n = 5 separate experiments for KO cells. (B) Phagocytic index were calculated [(% YG beads-positive cells) x (mean fluorescence intensity of YG beads-positive cells)] and shown as mean ± SD, with n = 4 separate experiments for wild type cells and n = 5 separate experiments for KO cells. Statistical significance was calculated using 2-way ANOVA followed by correction for false discovery rate and represented as '*' to compare the phagocytosis of -LPS KO to WT cells and as '#' to compare the LPS-treated KO vs. LPS-treated WT cells (*p < 0.05, **p < 0.01,#p < 0.05, ###p < 0.001). Negative controls for which phagocytosis was blocked by cytochalasin D treatment and by keeping cells on ice were performed with no positive cells obtained (data not shown here). [file Image_4.tif]
