# Supplementary material for: Immune response of BV-2 microglial cells is impacted by peroxisomal beta-oxidation
Source: Front Mol Neurosci. 2023 Dec 18;16:1299314. doi: 10.3389/fnmol.2023.1299314 (PMC10757945; doi:10.3389/fnmol.2023.1299314)

Western-blot Source Data

Table of Contents

- Caspase 1 slide no. 2
- Interleukin 1 beta slide no. 3
- NLRP3 slides no. 4
- CD36 slides no. 5-6
- FCGR2B slides no. 7
- MRC1 slides no. 8
- TLR2 slides no. 9
- TLR4 slides no. 10

Caspase 1

MW :
- 46 kDa (Pro-CASP1)
- 20 kDa (CASP1)

Primary antibody : Adipogen # AG-20B-0042

SDS-PAGE 12%
30 µg/load
PVDF Transfer

Samples serie S1 (21/10/21)
Mb « S1 C »

Samples serie S2 (27/10/21)
Mb « S2 Cb »

Mb selected for the publication

Samples serie S3 (28/10/21)
Mb «S3 C »

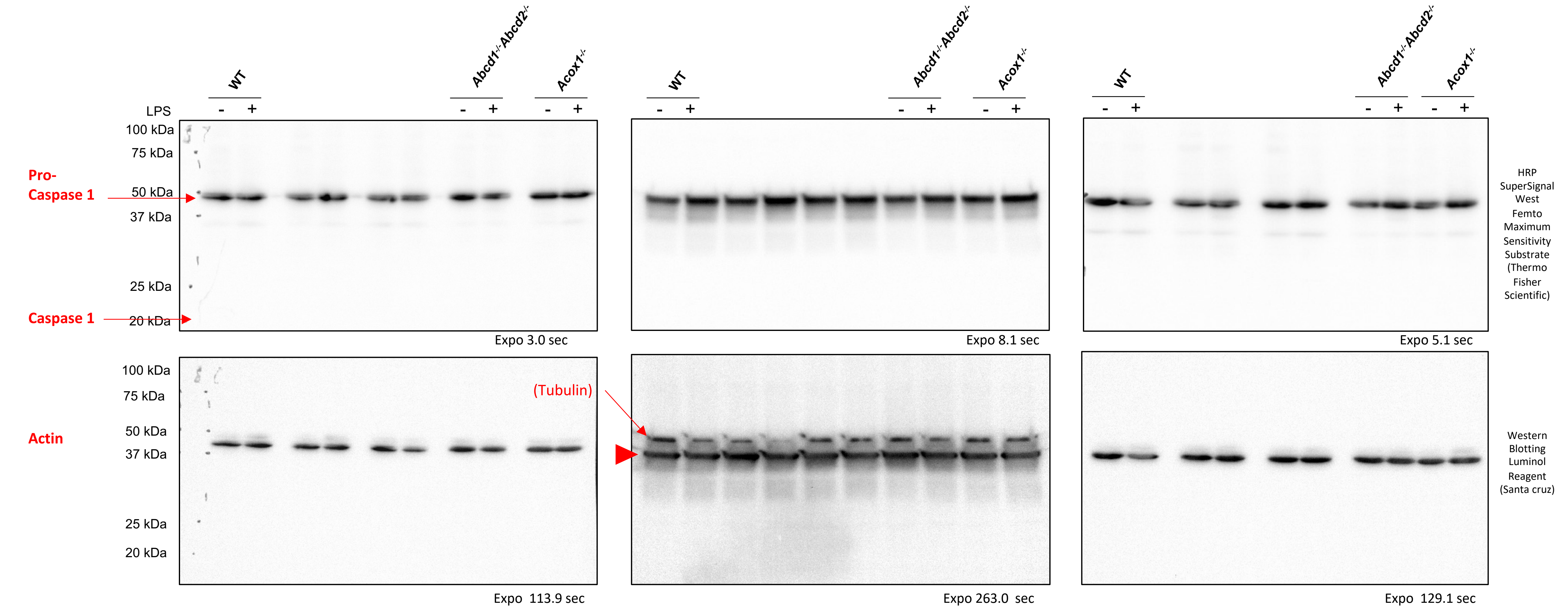

Interleukin 1 beta

MW :
- 31 kDa (Pro-IL1B)
- 17 kDa (IL1B)

Primary antibody : Gene Tex # GTX74034

SDS-PAGE 12%
30 µg/load
PVDF Transfer

Samples serie S1 (21/10/21)
Mb « S1 Ib »

Samples serie S2 (27/10/21)
Mb « S2 I »

Samples serie S3 (28/10/21)
Mb «S3 I »

Mb selected for the publication

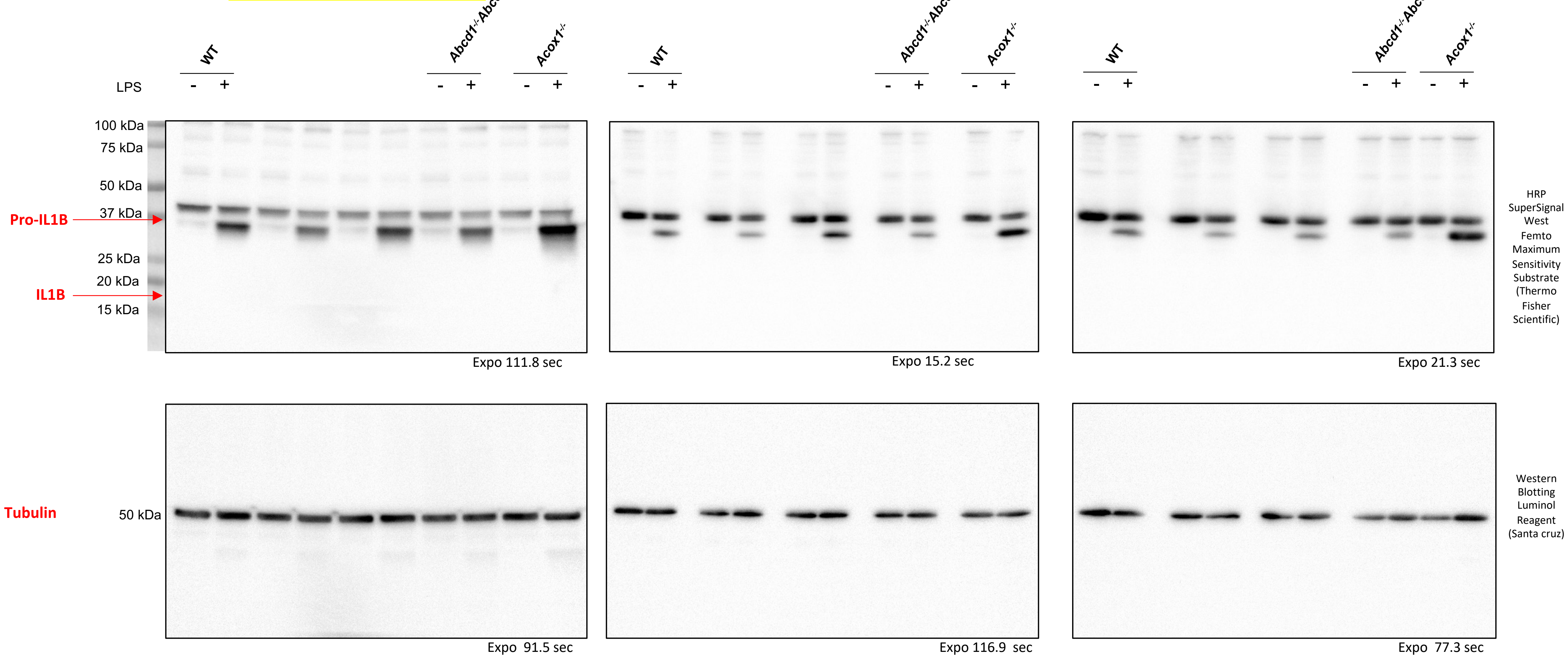

NLRP3

MW : ~ 110 kDa
Primary antibody : Adipogene # AG-20B-0014

SDS-PAGE gradient 4-15%
30 µg/load
PVDF Transfer

Samples serie S1 (21/10/21)
Mb « S1 T2bb »

Samples serie S2 (27/10/21)
Mb « S2 T2bb»

Samples serie S3 (28/10/21)
Mb «S3 T2bb »

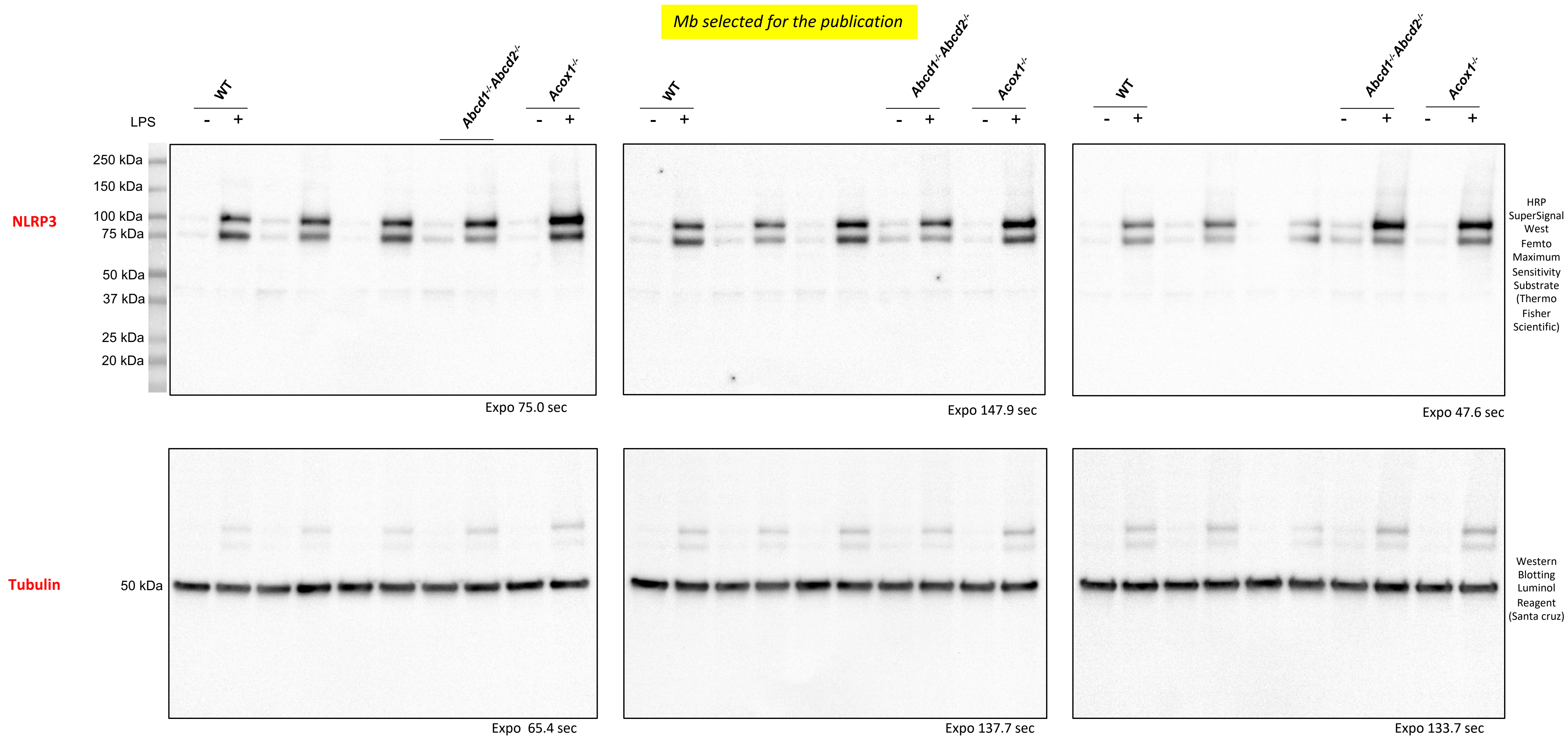

CD36

MW :

- 53 kDa (theoretical)
- 75-90 kDa (observed due to glycosylation)

Primary antibody : R&D Systems # AF2519

SDS-PAGE 4-15% gradient gels
30 µg/load
PVDF Transfer

Samples serie S1 (21/10/21)
Mb « S1 CD »

WT | $Abcd1^{-/-}Abcd2^{-/-}$ | $Acox1^{-/-}$

LPS - + | - + | - +

250 kDa
150 kDa
100 kDa
CD36
75 kDa
50 kDa
37 kDa
25 kDa

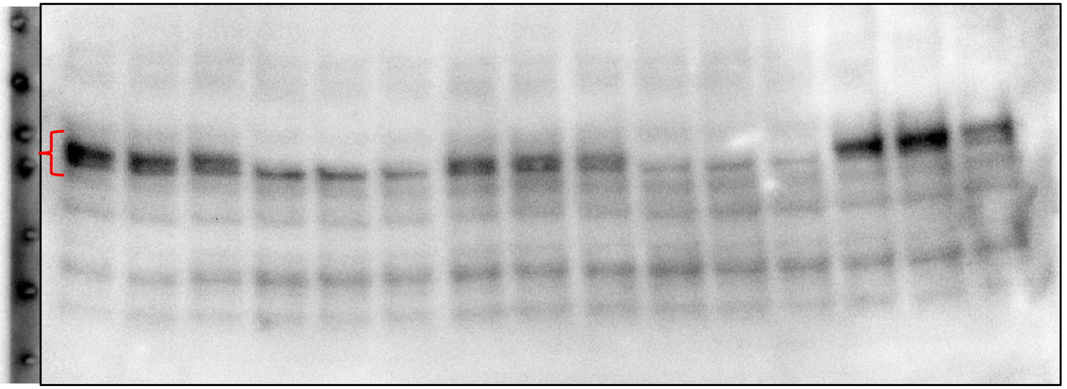

Expo 138.3 sec

Samples serie S2 (27/10/21)
Mb « S2 CD »

Mb selected for the publication

WT | $Abcd1^{-/-}Abcd2^{-/-}$ | $Acox1^{-/-}$

LPS - + | - + | - +

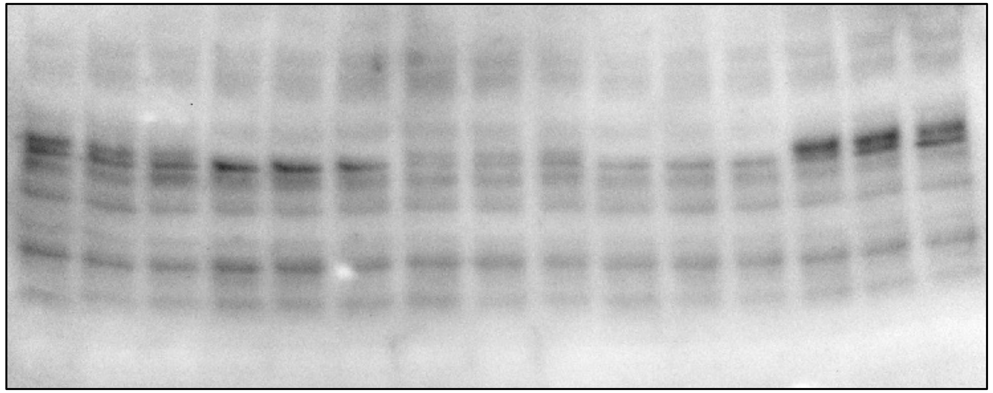

Expo 300.0 sec

HRP SuperSignal West Femto Maximum Sensitivity Substrate (Thermo Fisher Scientific)

Tubulin 50 kDa

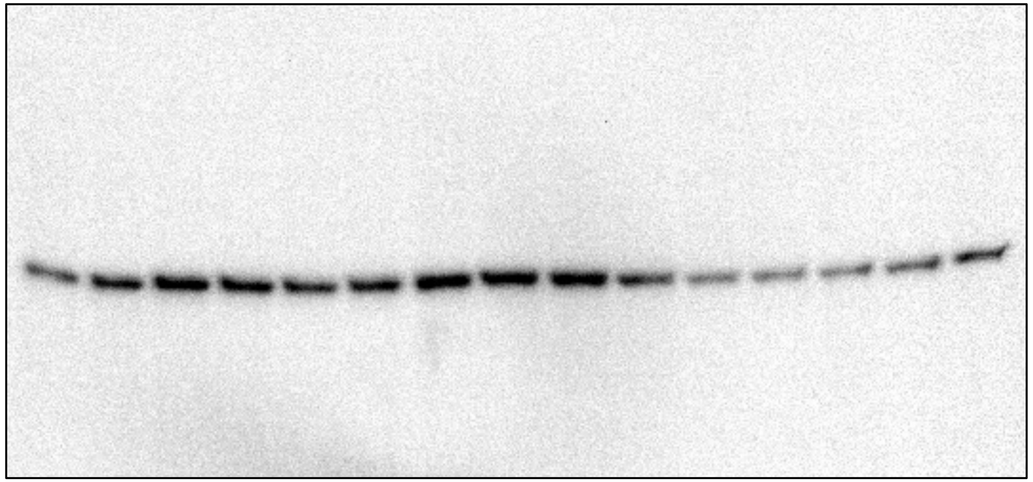

Expo 449.0 sec

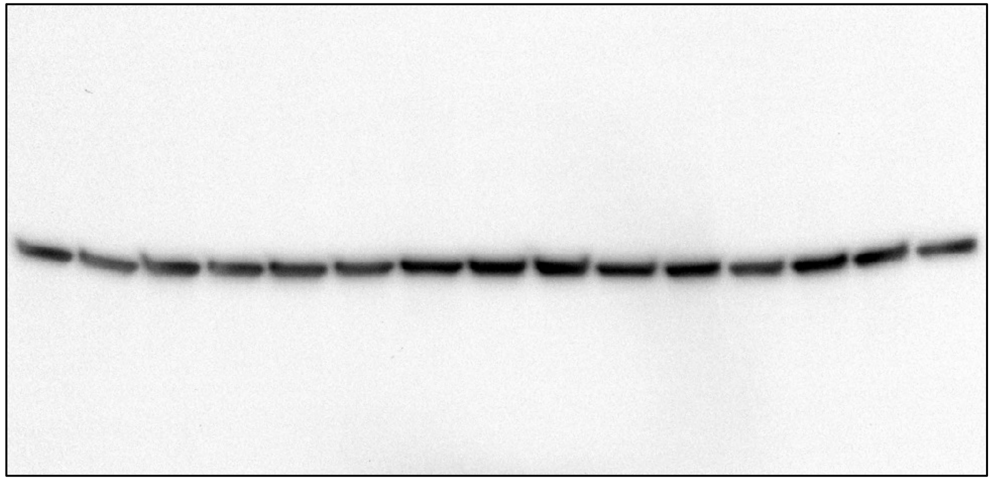

Expo 406.7 sec

Western Blotting Luminol Reagent (Santa cruz)

CD36

MW :

- 53 kDa (theoretical)
- 75-90 kDa (observed due to glycosylation)

Primary antibody : R&D Systems # AF2519

SDS-PAGE 4-15% gradient gels
30 µg/load
PVDF Transfer

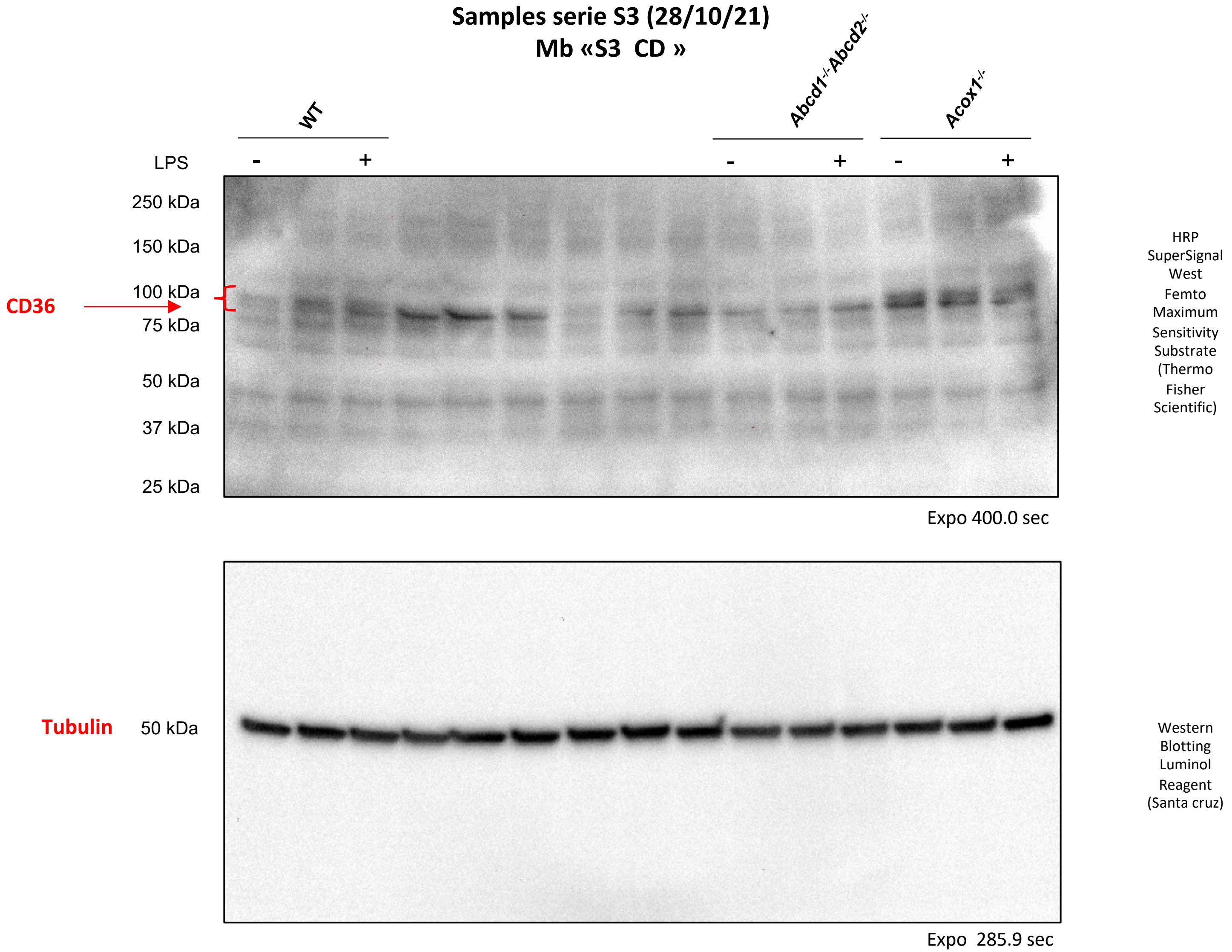

FCGRIIB

MW : ~ 70 kDa (observed)

Primary antibody : Cell Signaling # 96397

SDS-PAGE 4-15% gradient gels
30 µg/load
PVDF Transfer

Samples serie S1 (21/10/21)
Mb « S1 F »

Samples serie S2 (27/10/21)
Mb « S2 F »

Samples serie S3 (28/10/21)
Mb «S3 F »

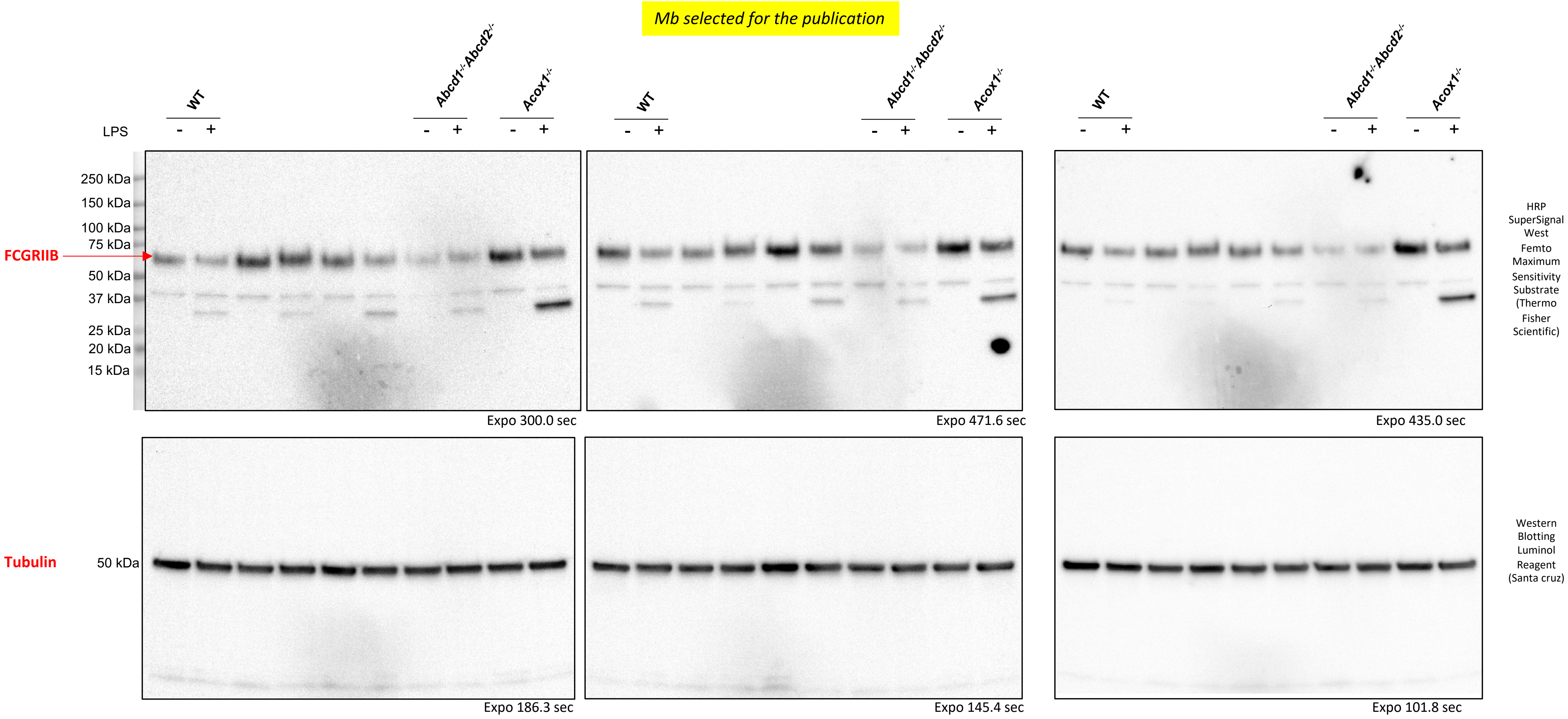

MRC1

MW : 165 kDa

Primary antibody : Abcam # 64693

SDS-PAGE 4-15% gradient gels
30 µg/load
PVDF Transfer

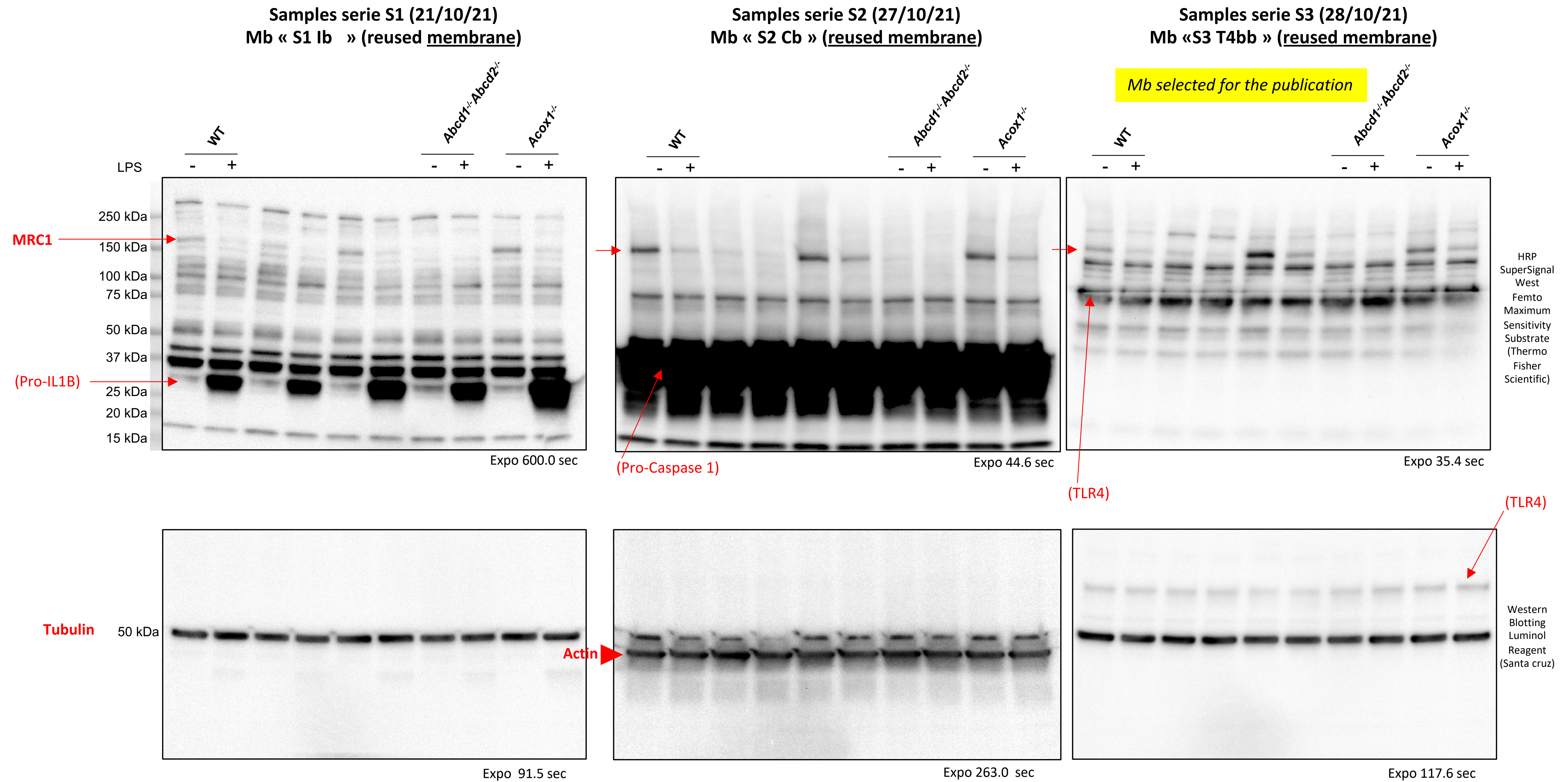

TLR2

MW : 90 kDa
Primary antibody : R&D Systems # AF1530

SDS-PAGE 4-15% gradient gels
30 µg/load
PVDF Transfer

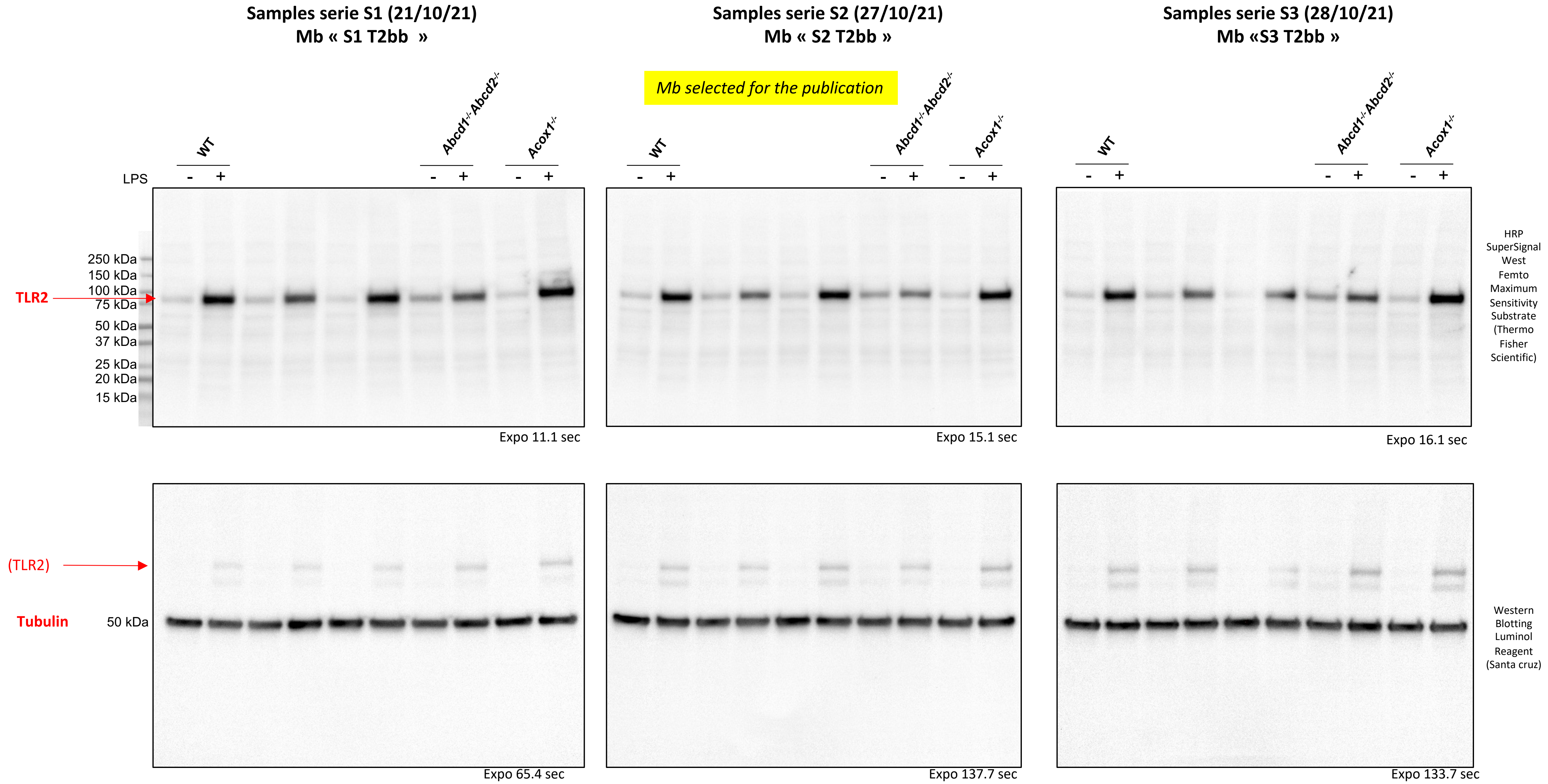

TLR4

MW : 96 kDa
Primary antibody : Santa Cruz # sc-293072

SDS-PAGE 8%
30 µg/load
PVDF Transfer

Samples serie S1 (21/10/21)
Mb « S1 T4b »

Samples serie S2 (27/10/21)
Mb « S2 T4b »

Samples serie S3 (28/10/21)
Mb «S3 T4bb » (4-15% gradient gel)

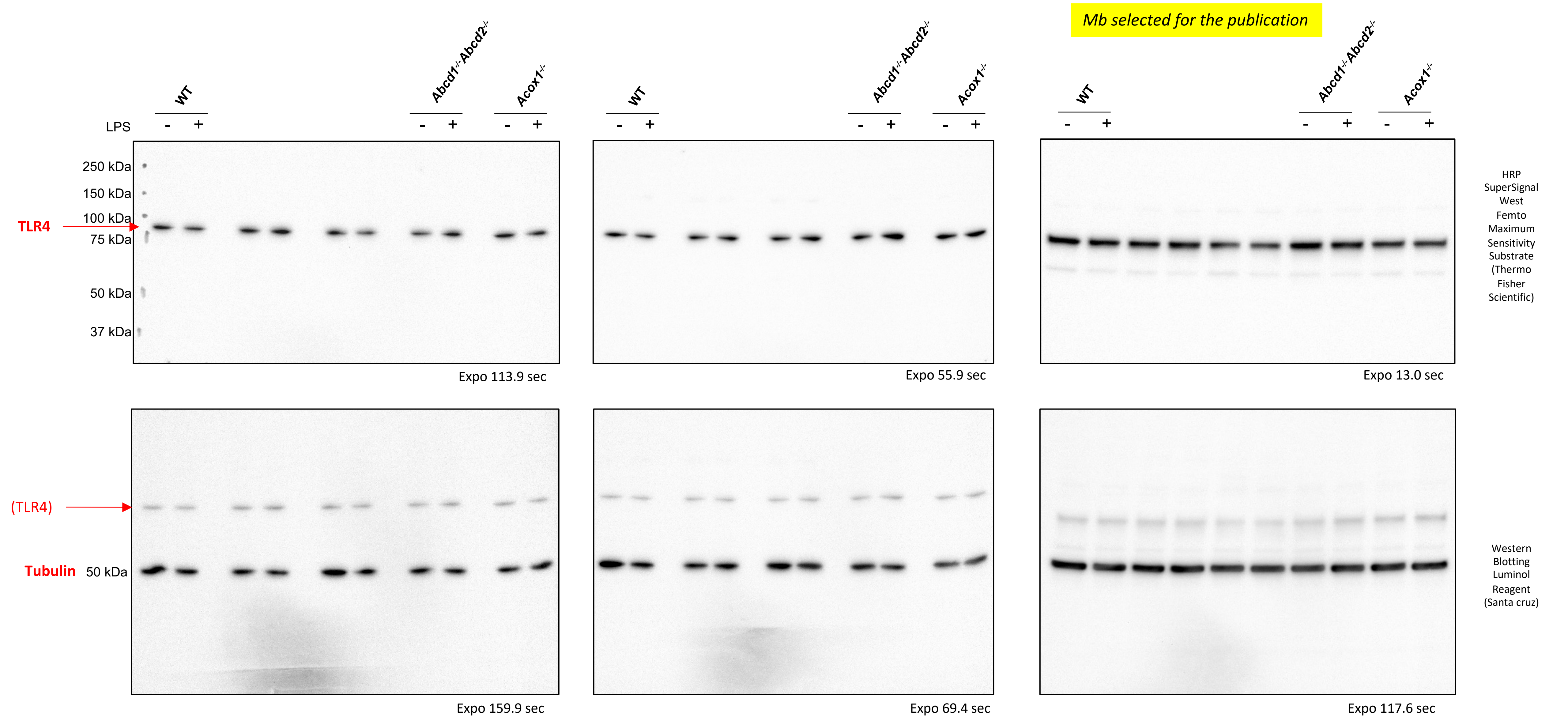

Supplement: DATA SOURCE — Uncropped blot for Western blot analysis of Caspase 1 (slide no. 2), Interleukin 1 beta (slide no. 3), NLRP3 (slide no. 4), CD36 (slides no. 5-6), FCGR2B slides no. 7), MRC1 (slide no. 8), TLR2 (slides no. 9), TLR4 (slide no. 10). [file Data_Sheet_1.pdf]
